# Cannabinoid receptors in the inflammatory cells of canine atopic dermatitis

**DOI:** 10.3389/fvets.2022.987132

**Published:** 2022-09-15

**Authors:** Roberto Chiocchetti, Giulia Salamanca, Margherita De Silva, Francesca Gobbo, Francesca Aspidi, Rodrigo Zamith Cunha, Giorgia Galiazzo, Claudio Tagliavia, Giuseppe Sarli, Maria Morini

**Affiliations:** ^1^Department of Veterinary Medical Sciences (UNI EN ISO 9001:2008), University of Bologna, Bologna, Italy; ^2^Faculty of Veterinary Medicine, Università degli Studi di Teramo, Località Piano D'Accio, Teramo, Italy

**Keywords:** cannabidiol, CB2R, GPR55, immunohistochemistry, TRPA1, TRPV1

## Abstract

**Background:**

Atopic dermatitis (AD) is one of the most common cutaneous inflammatory and pruritic diseases in dogs. Considering its multifactorial nature, AD can be a challenging disease to manage, and the therapeutic strategy must often be multimodal. In recent years, research has been moving toward the use of natural products which have beneficial effects on inflammation and itching, and no side effects. Cannabinoid receptors have been demonstrated to be expressed in healthy and diseased skin; therefore, one of the potential alternative therapeutic targets for investigating AD is the endocannabinoid system (ECS).

**Objective:**

To immunohistochemically investigate the expression of the cannabinoid receptor type 2 (CB2R), and the cannabinoid-related receptors G protein-coupled receptor 55 (GPR55), transient receptor potential vanilloid 1 (TRPV1) and ankyrin 1 (TRPA1) in mast cells (MCs), macrophages, dendritic cells (DCs), T cells, and neutrophils of the skin of dogs with AD.

**Animals:**

Samples of skin tissues were collected from eight dogs with AD (AD-dogs).

**Materials and methods:**

The immunofluorescent stained cryosections of the skins of 8 dogs with AD having antibodies against CB2R, GPR55, TRPV1, TRPA1 were semiquantitatively evaluated. The inflammatory cells were identified using antibodies against tryptase (mast cells), ionized calcium binding adaptor molecule 1 (IBA1) (macrophages/DCs), CD3 (T cells), and calprotectin (neutrophils). The proportions of MCs, macrophages/DCs, *T* cells, and neutrophils expressing CB2R, GPR55, TRPV1 and TRPA1 were evaluated.

**Results:**

The cells of the inflammatory infiltrate showed immunoreactivity (IR) for all or for some of the cannabinoid and cannabinoid-related receptors studied. In particular, MCs and macrophages/DCs showed CB2R-, GPR55-, TRPA1-, and TRPV1-IR; T cells showed CB2R-, GPR55- and TRPA1-IR, and neutrophils expressed GPR55-IR. Co-localization studies indicated that CB2R-IR was co-expressed with TRPV1-, TRPA1-, and GPR55-IR in different cellular elements of the dermis of the AD-dogs.

**Conclusions and clinical importance:**

Cannabinoid receptor 2, and cannabinoid-related receptors GPR55, TRPV1 and TRPA1 were widely expressed in the inflammatory infiltrate of the AD-dogs. Based on the present findings, the ECS could be considered to be a potential therapeutic target for dogs with AD, and may mitigate itch and inflammation.

## Introduction

Atopic dermatitis (AD) is one of the most common cutaneous inflammatory and pruritic diseases in dogs; it affects up to 27% of the canine population (1). Atopic dermatitis is associated with well-defined clinical signs and the overexpression of immunoglobulin IgE directed against environmental allergens, (s.c. extrinsic AD) (2–4) even if cases not due to IgE responses are known (s.c. intrinsic AD or atopic-like) (3). Several factors, in both humans and dogs, appear to contribute to skin inflammation and itching, such as increased exposure to pollutants, changes in dietary habits, stress, genetic factors, and cutaneous infections which predispose to the development of the disease.

The cells of the epidermis (keratinocytes) and the innate immune system play a critical role in AD, as shown not only in humans and rodents (5), but also in dogs (6). It has been shown that the skin of dogs with AD produces potent inflammatory mediators (7) and neurotrophins (8), which may be related to the hyperinnervation of the AD lesions (9). Pruritus, one of the most severe clinical signs of AD, is caused by a complex interface between pruritogenic molecules, keratinocytes, immunocytes, cutaneous nerve fibers, and the peripheral and central nervous systems (10).

Mast cells, strategically located at the sites directly interfacing with the external environment (11, 12), may release a variety of proinflammatory, vasoactive, and nociceptive mediators (13–15). However, in AD, keratinocytes and other inflammatory cell types, such as activated *T*-cells, macrophages, dendritic cells (DCs), Langerhans cells (LCs), basophils, and eosinophils may also display some abnormality (16, 17). In AD-related hypersensitivity a Th2-polarized lymphocyte response is activated by keratinocytes which produce cytokines (Interleukin [IL]-25 and IL-33), and thymic stromal lymphopoietin (TSLP) which leads to Th2 immune deviation (1, 8, 18, 19). Activated Th2 cells release IL-31 which stimulate itching by acting on IL-31 receptor A (IL-31RA) expressed on sensory nerve fibers and various immune cells, such as MCs, macrophages, DCs, eosinophils and basophils (20–26). Macrophages are also stimulated by inflammatory cytokines secreted by keratinocytes, such as granulocyte-macrophage colony-stimulating factor (GM-CSF), tumor necrosis factor-alpha (TNF-α), IL-6 and IL-2 (17, 27–29).

Over the past few years, research has been moving toward the use of natural products which have beneficial effects on inflammation and itching and, at the same time, do not have the side effects of more established therapies, such as those involving the use of glucocorticoids. One of the alternative potential therapeutic targets to investigate when AD is present is the endocannabinoid system (ECS). The ECS is composed of endogenous ligands (N-arachidonylethanolamine [anandamide, AEA] and 2-arachidonoyl glycerol [2-AG]), G-protein-coupled receptors (cannabinoid receptors 1 and 2 [CB1R and CB2R]) and enzymes aimed at degrading and recycling the ligands (30–32). The ECS contributes to the homeostasis of various organs and its dysregulation seems to be associated with several pathological conditions (31, 33–36).

The definition of the ECS has currently been expanded to also include several fatty acid derivatives—the so-called endocannabinoid-like mediators—as well as other cannabinoid-related receptors, such as the G protein-coupled receptors (GPRs), the transient receptor potential (TRP) channels, the nuclear peroxisome proliferator-activated receptors (PPARs), and the serotonin receptors in addition to the classic cannabinoid receptors and endocannabinoids (30, 37–40).

A recent study has demonstrated that cannabidiol (CBD), a non-psychotropic phytocannabinoid showing numerous health-related benefits, including anti-inflammatory and anti-anxiety properties (41, 42), may be useful in dogs with AD (1). Despite these promising clinical studies, there are still few studies dedicated to the histological localization of cannabinoid receptors in the canine inflammatory cells (43, 44). It is evident that knowing the cellular distribution of specific receptors is fundamental to understanding the action of a drug.

The role of the ECS in the keratinocytes of healthy dogs was recently analyzed, and the upregulation of the cannabinoid receptors (CB1R and CB2R) and cannabinoid-related receptors (GPR55, TRPV1, TRPA1; PPARα, serotonin 1A [5-HT1aR]) was evaluated in dogs with AD (45). In that study, CB2R, GPR55, TRPV1 and TRPA1 immunoreactivity was also observed on different cellular elements of the dermis. Therefore, the aim of the present study was to improve histological knowledge regarding the expression of cannabinoid and cannabinoid-related receptors in the inflammatory infiltrate of canine atopic dermatitis. In particular, the expression of the CB2R, GPR55, TRPV1, and TRPA1 was immunohistochemically investigated in MCs, macrophages, DCs, *T*-cells, and neutrophils, and the percentage of immunopositive inflammatory cells present on the total number of the same cell histotype was evaluated.

## Materials and methods

### Animals

#### Inclusion criteria

Eight client-owned dogs diagnosed with spontaneous AD, based on predefined diagnostic criteria (45) and on the exclusion of other causes of pruritus (flea bite, allergic dermatitis and adverse food reaction), were enrolled (Table 1). Cutaneous samples were collected from the AD-dogs on which no treatment had been made in the previous 6 months. Written client consent was obtained prior to the enrollment of all cases. The skin samples utilized in the current study derived from the same dogs included in a previous study (45).

**Table 1:** The clinical data of the dogs with atopic dermatitis (AD) enrolled in the present study.

**Dogs**	**Breed**	**Sex**	**Age**	**Pruritus visual analog scale (PVAS) (46)** **Canine atopic dermatitis extent and severity index (CADESI-4) (47)** **Skin area**
AD 1	Jack Russell	F^S^	7 yr	PVAS: 9/10 CADESI-4: 20 Groin
AD 2	French Bulldog	F	3 yr	PVAS: 8/10 CADESI-4: 48 Axilla (right and left)
AD 3	Cavalier King Charles Spaniel	M	8 yr	PVAS: 8/10 CADESI-4: 30 Groin
AD 4	Mixed breed	M	11 yr	PVAS: 6/10 CADESI-4: 20 Groin
AD 5	Akita Inu	M	4 yr	PVAS: 8/10 CADESI-4: 55 Axilla
AD 6	Golden Retriever	M	8 yr	PVAS: 9/10 CADESI-4: 60 Groin
AD 7	American Staffordshire Terrier	M	4 yr	PVAS: 8/10 CADESI-4: 58 Axilla
AD 8	French Bulldog	F	3 yr	PVAS: 8/10 CADESI-4: 32 Groin

F, female; F^s^, spayed female; M, male; M^n^, neutered male; NA, not available; yr, years.

#### Sample collection and processing

In the AD-dogs, a biopsy sample of skin lesions located in the ventral abdominal or axillary areas (Table 1) was collected using a sterile 8 mm biopsy punch. Sampling was carried out under local (2% lidocaine) anesthesia, using the same protocol for all dogs. The tissues from the AD-dogs were processed to obtain cryosections. The samples were fixed overnight in 4% paraformaldehyde in 0.1 M sodium phosphate buffer (pH 7.0) at +4°C. After being washed in phosphate-buffered saline (PBS 0.15 M NaCl in 0.01 M sodium phosphate buffer, pH 7.2), the tissues were immersed in PBS plus sodium azide 0.1% for 48 h (+4°C) and were then preserved in PBS–sodium azide 0.1% plus sucrose 30% (+4°C). All the samples were subsequently frozen in liquid nitrogen, and 14 μm-thick cryosections were obtained. The cryosections were hydrated in PBS and processed for histology and immunostaining.

#### Histopathology

The sections were hydrated in PBS for 10 mins and processed for histological staining with hematoxylin and eosin (H&E) following standard procedures. The sections were observed under an Eclipse E600 (Nikon, Shinjuku, Japan) optical microscope and evaluated following the criteria of Gross et al. (48). Images were acquired using an optical microscope (Eclipse E600; Nikon, Shinjuku, Japan) equipped with a USB 3.0 camera series “33” Imaging Source (cat. No. DFK 33UX264; Bremen, Germany).

### Immunofluorescence

The sections were hydrated in PBS and processed for immunostaining. To block non-specific bindings, the sections were incubated in a solution containing 20% normal donkey serum (Colorado Serum Co., Denver, CO, USA), 0.5% Triton X-100 (Sigma Aldrich, Milan, Italy, Europe) and bovine serum albumin (1%) in PBS for 1 h at room temperature (RT). The sections were incubated in a humid chamber overnight at RT with the antibodies directed against the CB2R and cannabinoid-related receptors (single immunostaining) or with a cocktail of primary antibodies (double immunostaining) (Table 2) diluted in 1.8% NaCl in 0.01 M PBS containing 0.1% sodium azide. After washing in PBS (3 × 10 mins), the sections were incubated for 1 h at RT in a humid chamber with the secondary antibodies (Table 3) diluted in PBS. The cryosections were then washed in PBS (3 × 10 min) and mounted in buffered glycerol at pH 8.6 with 4′,6-diamidino-2-phenylindole– DAPI (Santa Cruz Biotechnology, Santa Cruz, CA, USA).

**Table 2:** Primary antibodies used in the study.

**Primary antibody**	**Host**	**Code**	**Dilution**	**Source**
CB2R	Rabbit	ab45942	1:00	Abcam
CB2R	Mouse	sc-293188	1:50	Santa Cruz
CD3	Rat	CD3-12	1:40	Leucocyte's antigen laboratory, UC Davis
GPR55	Rabbit	NB110-55498	1:100	Novus Biol.
IBA1	Goat	NB100-1028	1:80	Novus Biol.
Calprotectin	Mouse	M0747 Clone MAC387	1:400	Dako
Tryptase	Rabbit	PAB070Ca01	1:80	Cloude-Clone
Tryptase	Mouse	Clone AA1	1:100	Agilent
Tryptase	Mouse	AM08408PU-N Clone 3H2643	1:50	Origene
Tryptase	Mouse	MAB070Ca21 Clone C13	1:50	Cloude-Clone
Tryptase	Mouse	sc-33676 Clone G3	1:50	Santa Cruz
TRPA1	Rabbit	ab58844	1:200	Abcam
TRPV1	Rabbit	ACC-030	1:200	Alomone

**Primary antibody Suppliers**: Abcam, Cambridge, UK; Agilent, Santa Clara, CA, USA; Alomone, Jerusalem, Israel; Cloude-Clone Corp., Huston, TX, USA; Dako Cytomation, Golstrup, Denmark; Novus Biologicals, Littleton, CO, USA; Origene Technologies, Rockville, MD, USA.

**Table 3:** Secondary antibodies used in the study.

**Secondary antibody**	**Host**	**Code**	**Dilution**	**Source**
Anti-mouse IgG Alexa-488	Donkey	A-21202	1:250	Thermo fisher
Anti-mouse IgG Alexa-594	Donkey	A-21203	1:500	Thermo fisher
Anti-goat 594	Donkey	ab150132	1:600	Abcam
Anti-rabbit 594	Donkey	ab150076	1:1000	Abcam
Anti-rabbit 488	Donkey	A-21206	1:1000	Thermo fisher

**Secondary antibody Suppliers:** Abcam, Cambridge, UK; Thermo Fisher Scientific, Waltham, MA USA.

The slides were examined using a Nikon Eclipse Ni microscope equipped with the appropriate filter cubes to differentiate the fluorochrome employed. The images were recorded using a Nikon DS-Qi1Nc digital camera and NIS elements software BR 4.20.01 (Nikon Instruments Europe BV, Amsterdam, Netherlands). The figure panels were prepared using Corel Draw (Corel Photo Paint and Corel Draw, Ottawa, ON, Canada).

### Specificity of the primary antibodies

#### Antibodies anti-cannabinoid receptor 2

The rabbit anti-CB2R antibody (ab45942) utilized in the present study had already been tested with Western blot (Wb) analysis on dog tissues (42) and tested for comparative purposes on rat tissues (49, 50). Another anti-CB2R antibody, raised in mice, was used in the current study to carry out the co-localization studies. Since the specificity of the mouse anti-CB2 antibody (sc-293188) had not already been tested on dog tissues, this antibody was co-localized with the rabbit anti-CB2 antibody in a double-staining protocol. Both the anti-CB2R antibodies were co-localized in keratinocytes and blood vessels (Supplementary Figure 1). In the dermal cells, the immunostaining obtained with the antibody raised in mice (sc-293188) was brighter than that raised in rabbits (ab45942).

#### Antibodies anti-cannabinoid-related receptors GPR55, TRPA1, and TRPV1

In the present study, the anti-human GPR55 (NB110-55498) antibody was used, the specificity of which was tested on dog tissues using Western blot (Wb) analysis (49).

The immunogen used to obtain the anti-TRPA1 antibody was the EKQHELIKLIIQKME peptide, corresponding to amino acids 1,070–1,085 of rat TRPA1. The homology between the full amino acid sequences of the dog and rat TRPA1 was 82.29%, and correspondence with the specific sequence of the immunogen was 100%; therefore, the anti-TRPA1 antibody should also recognize the same receptor in the dog.

The immunogen of the anti-TRPV1 antibody was the (C)EDAEVFK DSMVPGEK [824–838] peptide of rat TRPV1. The homology between the specific amino acid sequences of the dog and the rat immunogens was 87.51%. The specificity of the rabbit anti-TRPV1 antibody, which has recently been tested on the canine nervous system (49), was tested using a preadsorption test on the canine skin (45).

The homologies of the canine receptors studied in the dogs (CB2R, GPR55, TRPV1, and TRPA1) were verified using the ‘alignment' tool available on the Uniprot database (www.uniprot.org) and the BLAST tool of the National Center for Biotechnology Information (NCBI) (www.ncbi.nlm.nih.gov).

#### Anti-mast cells tryptase antibodies

The skin tissues were processed for cryosectioning to avoid any thermal and chemical modifications of the receptors studied and the background and self-marking of the tissues which are unfortunately often observable in paraffin-embedded tissues. Unexpectedly, some difficulties in MC identification were encountered, and it was necessary to test more anti-tryptase antibodies. The only antibody, among those tested, capable of effectively identifying MCs in the cryosections was the rabbit anti-tryptase antibody (PAB070Ca01), raised against the tryptase of dogs (43); its specificity was also tested in the current study by combining immunohistochemical staining in association with toluidine blue as a counterstain (44) (Supplementary Figure 2). In the present study, the rabbit anti-tryptase antibody was used in co-localization with the mouse anti-CB2R antibody.

However, since the antibodies directed against GPR55, TRPV1, and TRPA1 were raised in rabbits, co-localization studies with the rabbit anti-tryptase antibody were not possible. Therefore, the expression of the cannabinoid-related receptors in MCs was evaluated using anti-tryptase antibodies raised in mice. Of the four different mouse anti-tryptase antibodies tested (Table 3), only clone 3H2643 was capable of immunolabelling the MCs in the cryosections whereas all the antibodies were capable of identifying the MCs in the paraffin embedded sections (observation of Dr. Gobbo). The co-localization between the dog-specific rabbit anti-tryptase antibody (PAB070Ca01) and the mouse anti-tryptase antibody (clone 3H2643) on the cryosections showed that MCs co-expressed the same tryptase-IR. In addition, only a few other cells (likely basophils) were immunolabelled by the clone 3H2643, and were not recognized by the PAB070Ca01 antibody (data not shown).

#### Marker for macrophages and dendritic cells

To identify macrophages, the anti-ionized calcium binding adapter molecule 1 (IBA1) antibody was used (43). Since in the dog skin, IBA1-IR is also expressed by DCs (51) and, in the current study, no specific markers to differentiate DCs from macrophages were used, the term “IBA1 immunoreactive cells” will refer to both cellular types (macrophages/DCs). It was also observed that IBA1 immunoreactive DCs were dispersed among the keratinocytes of the basal layer of the epidermis, supporting the fact that the IBA1 marker is also expressed by dog skin DCs (data not shown). The intraepithelial DCs were more properly defined as Langerhans cells, given their specific site.

#### Marker for neutrophils

An antibody anti-CAL (clone MAC387), a complex of the mammalian proteins S100A8 and S100A9 (S100A8/A9), was used as a marker for granulocytes/monocytes/macrophages. Since the co-localization between anti-CAL and -IBA1 antibodies did not show any co-localization (Supplementary Figure 3), it is plausible to think that, at least in the skin, the anti-calprotectin (CAL) (MAC387) antibody mainly recognizes neutrophils instead of macrophages (52). The observation of Dapi labeled multilobed nuclei confirmed that the CAL immunoreactive cells were neutrophils. This evidence was supported by the observation of Kerkhoff et al. (53) who showed that S100A8/A9 comprises ~30 to 60% of all cytosolic proteins in neutrophils and only 1–5% of all monocyte cytosolic proteins, and that macrophages express and release significantly less S100A8/A9 than do monocytes. In addition, there was evidence that CAL expression was lost during the maturation of canine macrophages (54, 55), and that it was more suited to assessing migrating monocytes and early stages of macrophage maturation rather than the resident macrophage population (56).

#### Marker for T lymhpocytes

To identify *T*-cells, the dog-specific anti-cluster of differentiation 3 (CD3) antibody was used (57). A recent study has shown that canine CD3 might also be expressed by Th2 cells (26) which play a pivotal role in AD pathogenesis.

### Specificity of the secondary antibodies

The specificity of the secondary antibodies was tested by applying them after omission of the primary antibodies. No stained cells were detected after omitting the primary antibodies.

### Semiquantitative and quantitative analysis

The immunoreactivity of the antibodies was evaluated, and its cellular localization (nuclear, membranous, cytoplasmic) was reported. The intensity of the expression was evaluated semiquantitatively in pictures acquired using the same exposure times, as faint, moderate and bright. The proportions of dermal cells which were immunoreactive for the markers of MCs (tryptase), macrophages/DCs (IBA1), *T*-cells (CD3), and neutrophils (CAL), and which were also immunoreactive for one of the cannabinoid receptors studied were determined by examining fluorescently labeled, double-stained preparations. Digital images of areas of the sections with a high density of inflammatory cells, located just below the dermal–epidermal junction, were acquired at x40 magnification. The cells were first located by the presence of a fluorophore which labeled one antigen; the filter was then switched to determine whether or not the cells were labeled for a second antigen, located with a fluorophore of a different color. In this way, the proportions of inflammatory cells labeled for pairs of antigens were determined. For each staining combination, sections of the skin were used from three *to five* different AD-dogs. At least 30 cells from each animal were counted for each inflammatory cells marker. The percentage of cells which were immunoreactive for a particular marker (tryptase, IBA1, CD3, and CAL) and which were also immunoreactive for a second marker (CB2, GPR55, TRPV1, TRPA1) was calculated and expressed as mean ± standard deviation, with *n* being the number of AD-dogs considered.

## Results

### Histopathology

All eight samples were histopathologically diagnosed as chronic hyperplastic mixed perivascular dermatitis, consistent with the inflammatory pattern of canine AD. Specifically, the skin showed moderate to severe, focal to diffuse hyperplasia in the superficial (hyperkeratosis) and the suprabasal (acanthosis) layers of the epidermis; in the dermis, superficial perivascular to interstitial mild mixed inflammatory infiltrates (lymphocytes, histiocytes, mast cells, plasma cells and few eosinophils) were detected. In four of the eight cases, a periannesial inflammatory infiltrate, predominantly neutrophilic, was also observed (multifocal moderate pyogranulomatous dermatitis, presumably an infection secondary to chronic AD).

### Cannabinoid receptors in tryptase immunoreactive mast cells

**Figure 1:**
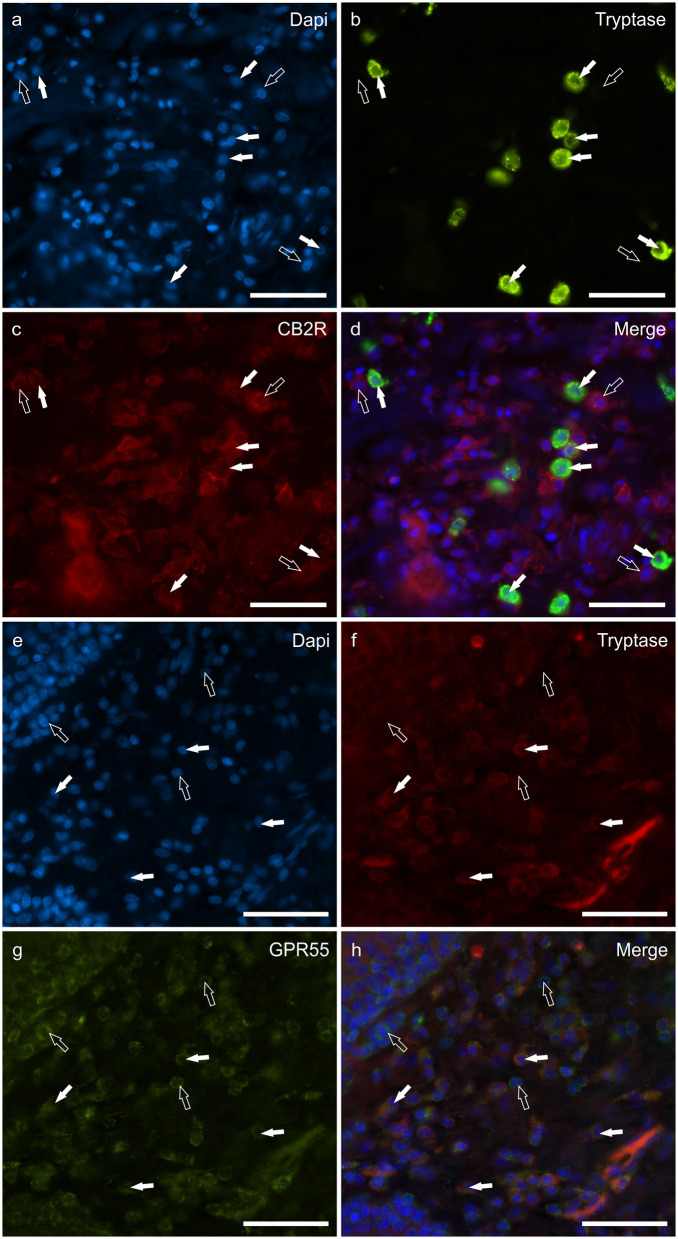
Photomicrographs of the cryosections of the skin of dogs with atopic dermatitis (AD) showing tryptase immunoreactive mast cells (MCs) expressing cannabinoid receptor 2 (CB2R) **(a–d)** and G-protein coupled receptor 55 (GPR55) **(e–h)** immunoreactivity (IR). The white arrows indicate the Dapi-labeled nuclei of tryptase positive MCs co-expressing CB2R- **(c)** and GPR55-IR **(g)**. The open arrows indicate some tryptase negative cells of the dermis which expressed CB2R- **(c)** and GPR55-IR **(g)**. Bar: 50 μm.

**Figure 2:**
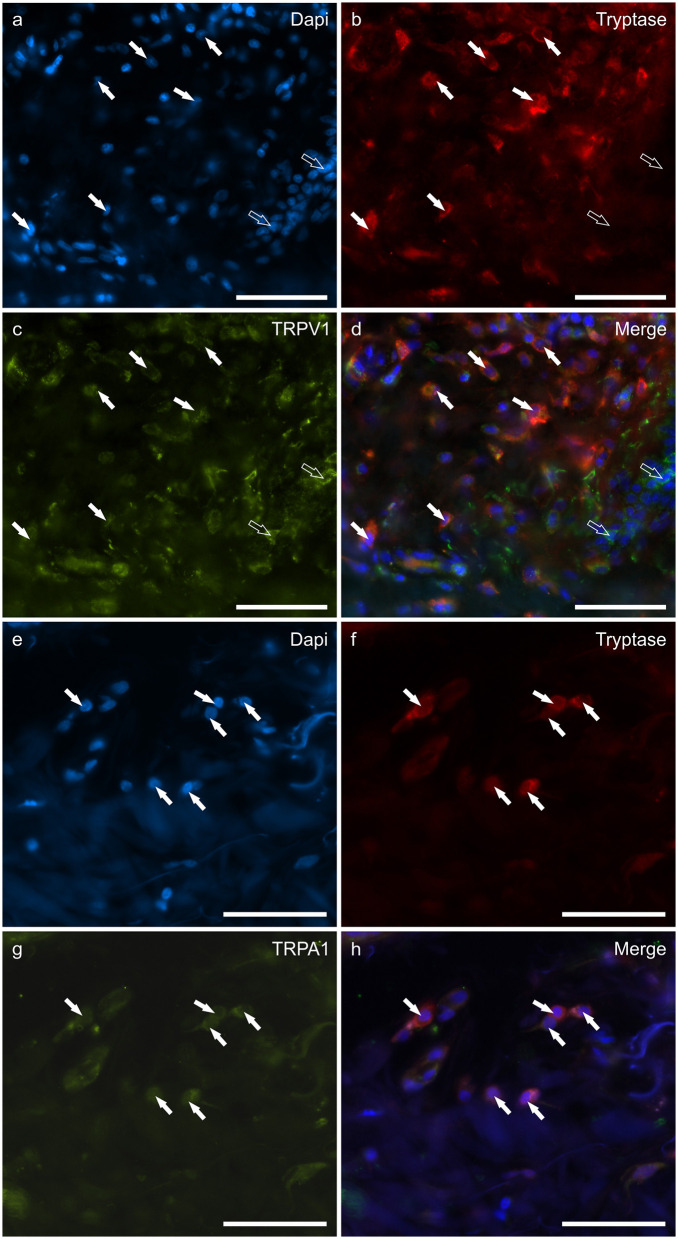
Photomicrographs of the cryosections of the skin of dogs with atopic dermatitis (AD) showing tryptase immunoreactive mast cells (MCs) expressing transient receptor potential vanilloid 1 (TRPV1) **(a–d)** and transient receptor potential ankyrin 1 (TRPA1) **(e–h)** immunoreactivity (IR). The white arrows indicate the Dapi-labeled nuclei of tryptase positive MCs co-expressing TRPV1- **(c)** and TRPA1-IR **(g)**. The open arrows indicate some tryptase negative cells of the dermis which expressed TRPV1-IR **(c)**. Bar: 50 μm.

The mast cells were immunoreactive for all the receptors investigated (CB2R, GPR55, TRPV1, and TRPA1). Approximately a quarter of the MCs expressed moderate CB2-IR in the cell membrane and the cytoplasm (26 ± 17%, 106/428 cells, *n* = *4*) (Figures 1a–d). A large proportion of MCs showed bright cytoplasmic GPR55-IR, albeit with large individual variations (65 ± 38%, 75/168 cells, *n* = *4*) (Figures 1e–h). The mast cells showed moderate cytoplasmic TRPV1-IR (74 ± 20%, 172/229 cells, *n* = *4*) (Figures 2a–d) and TRPA1-IR (66 ± 40%, 166/302 cells, *n* = *5*) (Figures 2e–h).

### Cannabinoid receptors in IBA1 immunoreactive macrophages/DCs

**Figure 3:**
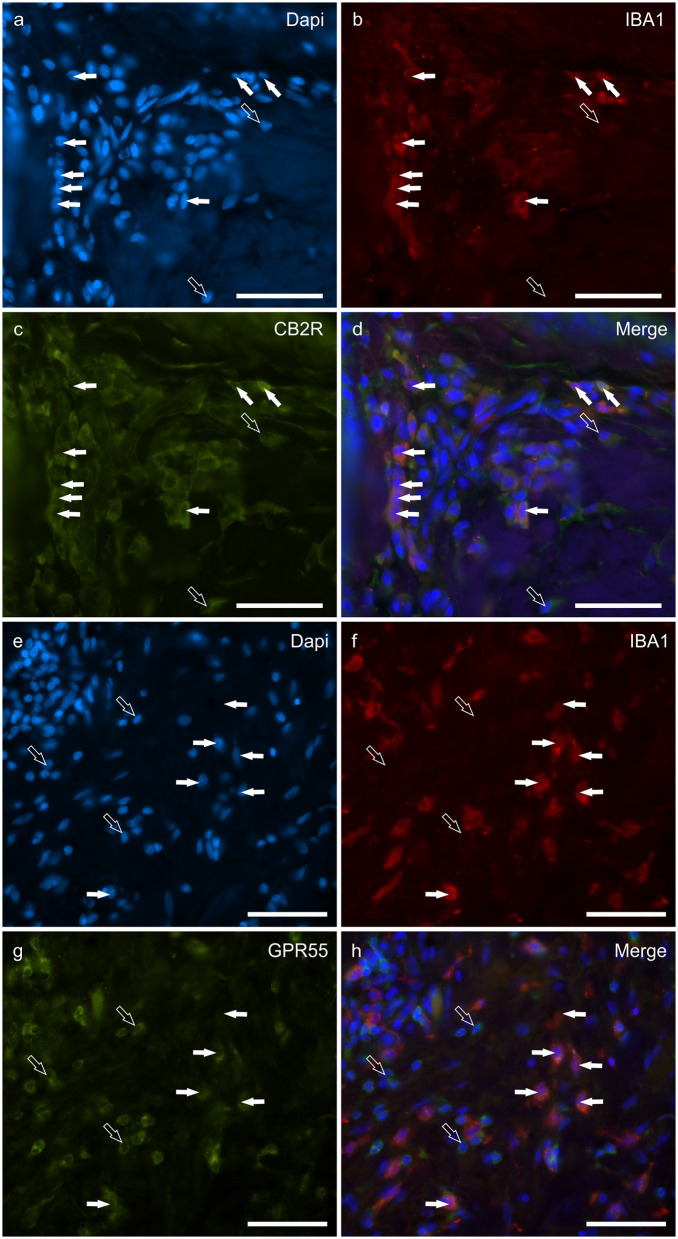
Photomicrographs of the cryosections of the skin of dogs with atopic dermatitis (AD) showing IBA1 immunoreactive cells (macrophages and dendritic cells) expressing cannabinoid receptor 2 (CB2R) **(a–d)** and G-protein coupled receptor 55 (GPR55) **(e–h)** immunoreactivity (IR). The white arrows indicate the Dapi-labeled nuclei of IBA1 positive cells co-expressing CB2R- **(c)** and GPR55-IR **(g)**. The open arrows indicate some IBA1 negative cells of the dermis which expressed CB2R- **(c)** and GPR55-IR **(g)**. Bar: 50 μm.

**Figure 4:**
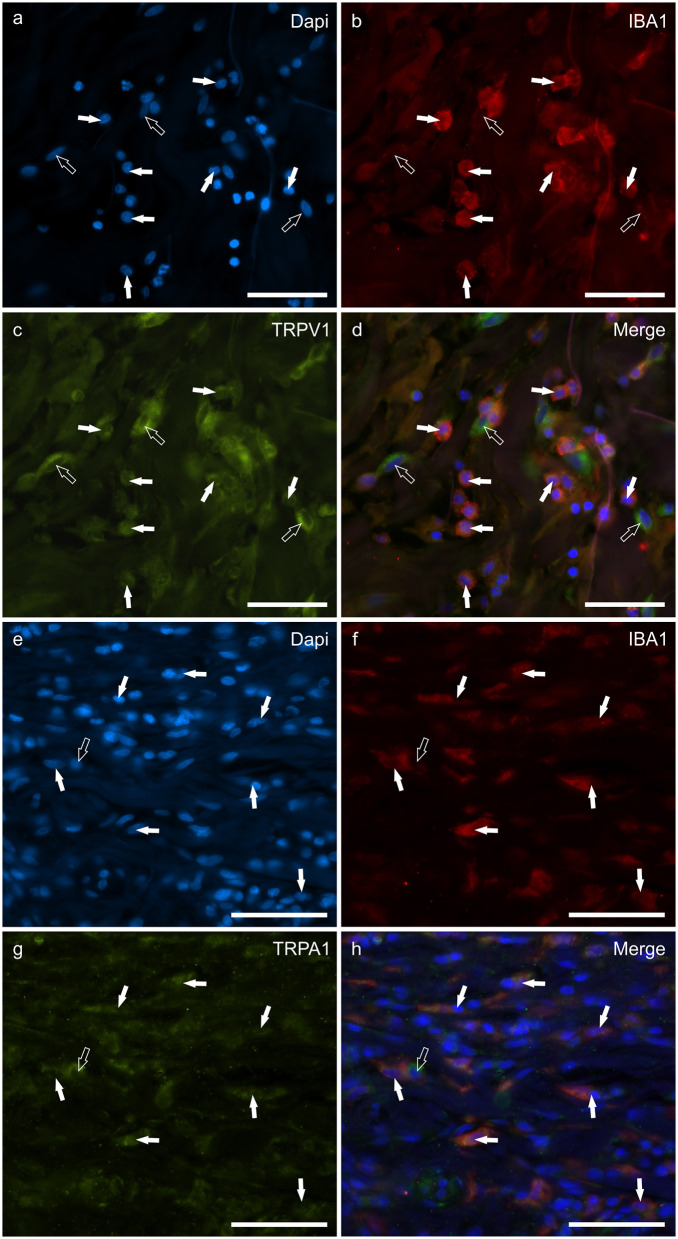
Photomicrographs of the cryosections of the skin of a dog with atopic dermatitis (AD) showing IBA1 immunoreactive cells (macrophages and dendritic cells) expressing transient receptor potential vanilloid 1 (TRPV1) **(a–d)** and ankyrin 1 (TRPA1) immunoreactivity (IR) **(e–h)**. The white arrows indicate the Dapi-labeled nuclei of IBA1 positive cells co-expressing TRPV1-IR **(c)** and TRPA1-IR **(g)**. The open arrow indicates one IBA1 negative cell which expressed TRPV1- **(c)** and TRPA1-IR **(g)**. Bar: 50 μm.

Macrophages/DCs were consistently observed in the derma of the AD-dogs, although they had a different distribution and density. Large proportions of IBA1 immunoreactive cells, which were often grouped in close proximity to the blood vessels, showed immunoreactivity for CB2R, GPR55, TRPV1, and TRPA1. In particular, the cell membrane and the cytoplasm of the macrophages/DCs expressed bright CB2R-IR (91 ± 16%, 294/307 cells, *n* = *4*) (Figures 3a–d) and moderate GPR55-IR (68 ± 18%, 253/390 cells, *n* = *3*) (Figures 3e–h). In the subepidermal connective tissue, large IBA1 immunoreactive cells were often intermingled with small GPR55 immunoreactive (and IBA1 negative) cells (likely lymphocytes). The macrophages/DCs also showed cytoplasmic moderate TRPV1- (80 ± 16%, 191/241 cells, *n* = *3*) (Figures 4a–d) and faint TRPA1-IR (81 ± 14%, 224/265 cells, *n* = *4*) (Figures 4e–h).

### Cannabinoid receptors in calprotectin immunoreactive neutrophils

**Figure 5:**
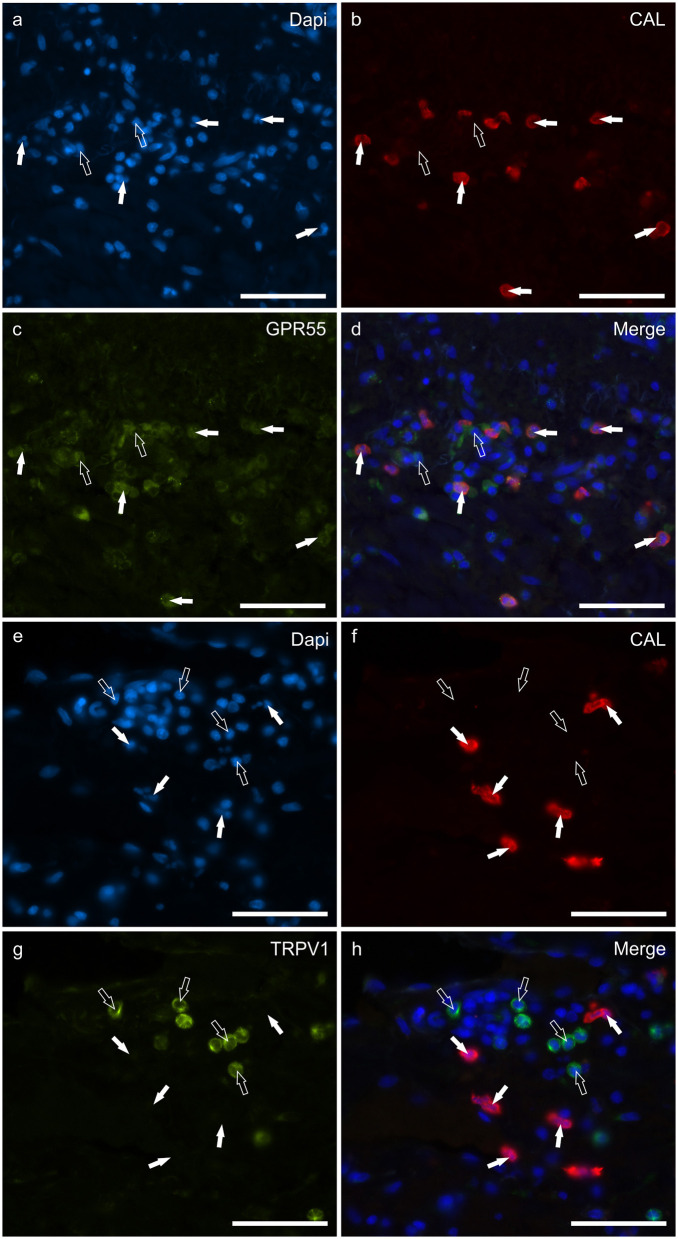
Photomicrographs of the cryosections of the skin of dogs with atopic dermatitis (AD) showing Calprotectin (CAL) immunoreactive cells expressing G-protein coupled receptor 55 (GPR55) **(a–d)** and transient receptor potential vanilloid 1 (TRPV1) immunoreactivity (IR) **(e–h)**. **(a–d)** The white arrows indicate the Dapi-labeled nuclei of CAL positive cells co-expressing GPR55-IR **(c)**. The open arrows indicate some CAL negative cells expressing GPR55-IR **(c)**. **(e–h)** The white arrows indicate the Dapi-labeled nuclei of the CAL positive cells which were TRPV1 negative **(g)**. The open arrows indicate some TRPV1-IR cells which were CAL negative **(g)**. Bar: 50 μm.

Round, and often irregularly-shaped cells, expressed CAL-IR in the derma of the AD-dogs; the density of the CAL immunoreactive cells was variable depending on the AD-dog skin samples. Small groups of few CAL positive cells were scattered in proximity of the epidermis, or were intravascular. A large proportion (77 ± 8%, 84/112 cells, *n* = *3*) of CAL positive neutrophils showed moderate cytoplasmic GPR55-IR (Figures 5a–d). None of the CAL immunoreactive neutrophils were TRPV1 (0/162 cells, *n* = 3) (Figures 5e–h) or TRPA1 (0/150 cells, n = 3) immunoreactive (data not shown). Due to the fact that the anti-CB2 antibody was also raised in mice, co-localization between the anti-CAL and the -CB2R antibodies was not carried out. However, since the co-localization study between the anti-CB2R and the -GPR55 antibodies showed that the inflammatory cells expressed both the markers (Supplementary Figure 4), it is plausible that CAL immunoreactive cells may also express CB2R-IR.

### Cannabinoid receptors in CD3 immunoreactive *T*-cells

**Figure 6:**
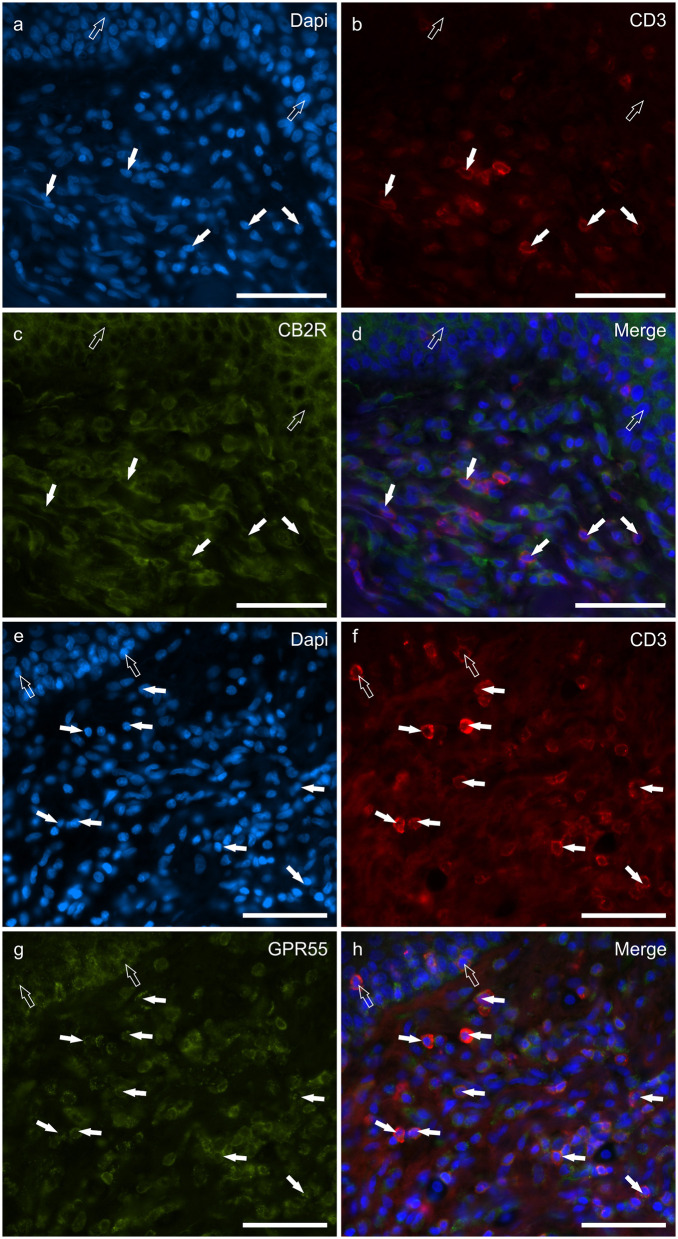
Photomicrographs of the cryosections of the skin of dogs with atopic dermatitis (AD) showing CD3 immunoreactive T lymphocytes expressing cannabinoid receptor 2- (CB2R) **(a–d)** and G-protein coupled receptor 55- (GPR55) **(e–h)** immunoreactivity (IR) **(e–h)**. **(a–d)** The white arrows indicate the Dapi-labeled nuclei of some CD3 immunoreactive T lymphocytes co-expressing CB2R-IR. The open arrows indicate the nuclei of the keratinocytes expressing CB2R-IR **(c)**. **(e–h)** The white arrows indicate the dapi-labeled nuclei of some CD3 immunoreactive T lymphocytes co-expressing GPR55-IR **(g)**. The open arrows indicate the nuclei of two intraepithelial T lymphocytes co-expressing CD3- **(f)** and GPR55-IR **(g)**. Bar: 50 μm.

**Figure 7:**
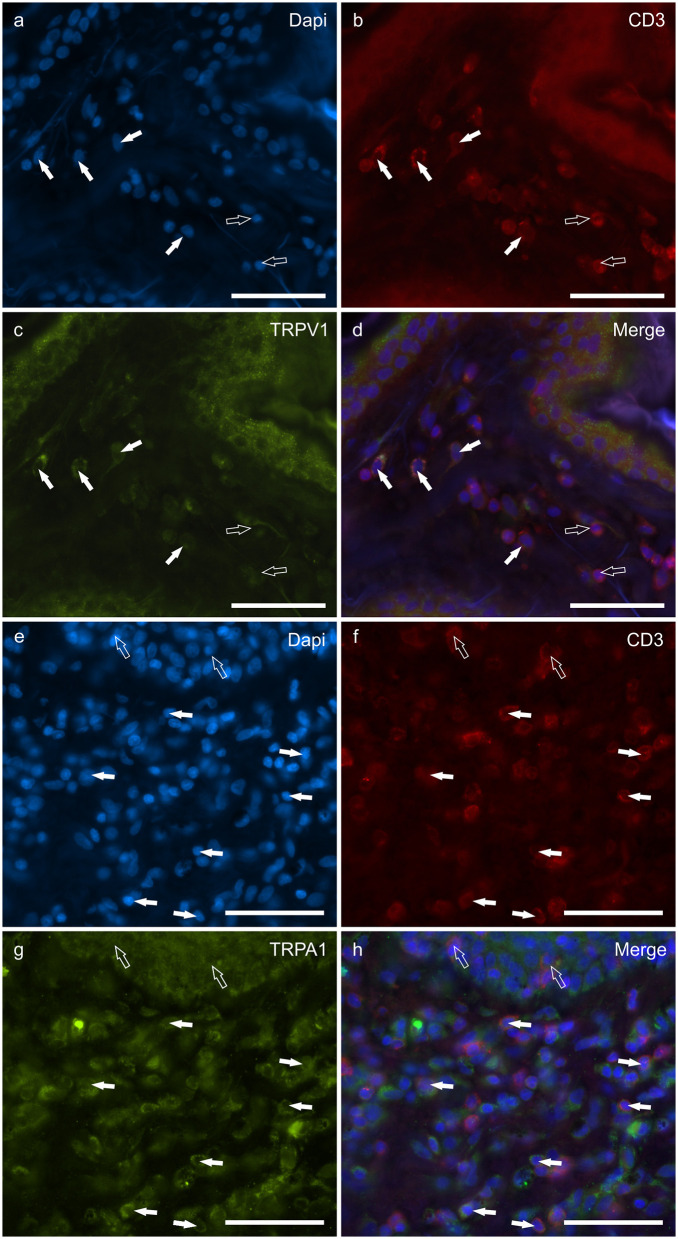
Photomicrographs of the cryosections of the skin of dogs with atopic dermatitis (AD) showing CD3 immunoreactive T lymphocytes expressing transient receptor potential vanilloid 1 (TRPV1) **(a–d)** and transient receptor potential ankyrin 1 (TRPA1) **(e–h)** immunoreactivity (IR). **(a–d)** The white arrows indicate the dapi-labeled nuclei of some CD3 immunoreactive T lymphocytes co-expressing TRPV1-IR **(C)**. The open arrows indicate the nuclei of CD3-IR cells which were TRPV1 negative. **(e–h)** The white arrows indicate the Dapi-labeled nuclei of some CD3 immunoreactive T lymphocytes co-expressing TRPA1-IR **(g)**. The open arrows indicate the nuclei of two intraepithelial CD3 immunoreactive lymphocytes **(f)**. Bar: 50 μm.

T lymphocytes were well represented in the derma of all the AD-dogs considered. The CD3-IR was more expressed in those *T*-cells scattered within the epithelial cells. However, it was shown that the CD3 immunoreactive cells showed immunoreactivity for CB2R, GPR55, TRPV1 and TRPA1. In particular, the cell membrane and the cytoplasm of the *T*-cells expressed faint-to-moderate CB2R-IR (28 ± 11%, 137/278 cells, *n* = *5*) (Figures 6a–d) and bright GPR55-IR (90 ± 15 %, 289/352 cells, *n* = *4*) (Figures 6e–h). Many other small CD3 negative cells, likely B lymphocytes, expressed GPR55-IR, as recently shown in the dog intestine (43). The cell membrane and the cytoplasm of the *T*-cells expressed faint TRPV1 (30 ± 19%, 49/159 cells, *n* = *4*) (Figures 7a–d) and moderate TRPA1-IR (52 ± 15%, 48/112 cells, *n* = *4*) (Figures 7e–h).

**Figure 8:**
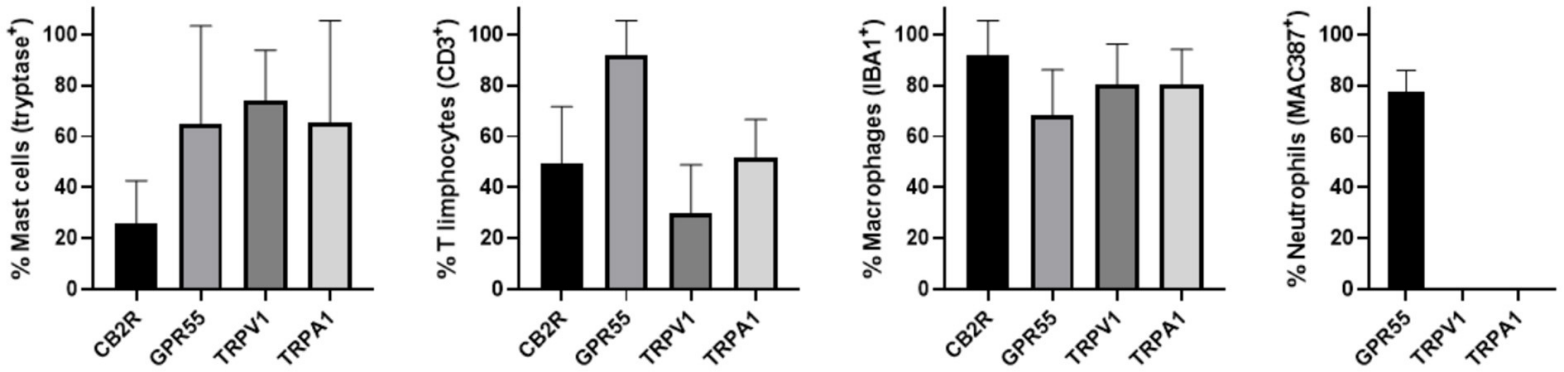
Percentage of mast cells, T limphocytes, macrophages/dendritic cells and neutrophils that expressed CB2R, GPR55, TRPV1 and TRPA1. Mast cells expressing tryptase were immunoreactive for CB2R, GPR55, TRPV1, and TRPA1. T limphocytes expressing CD3 were immunoreactive for CB2R, GPR55, TRPV1, and TRPA1. Macrophages/dendritic cells expressing IBA1 were immunoreactive for CB2R, GPR55, TRPV1, and TRPA1. Neutrophils expressing MAC387 were immunoreactive only for GPR55. Data are presented as Mean (±SD).

The semi-quantitative evaluation of the intensity of the immunolabelling of the cannabinoid and cannabinoid-related receptors studied in the skin of the AD-dogs is reported in Table 4. The data related to the distribution of the cannabinoid receptors in MCs, macrophages/DCs, *T*-cells and neutrophils are graphically represented in Figure 8.

**Table 4:** Semiquantitative evaluation of the density of CB2R, GPR55, TRPV1, and TRPA1 immunoreactivity in different cellular elements of the skin of dogs with atopic dermatitis (AD-dogs).

**Receptors**	**Mast cells (tryptase)**	**Macrophages and dendritic cells (IBA1)**	***T*-cells (CD3)**	**Neutrophils (CAL)**
CB2R	++	+++	+	^NA^
GPR55	+++	++	+++	++
TRPV1	++	++	+	–
TRPA1	++	+	++	–

C, cytoplasmic; M, membrane; NA, not applied. Immunoreactive cells were graded as: –, negative; +, faint; ++, moderate; +++, bright.

Co-localization studies showed that CB2R-IR was co-expressed by subsets of GPR55- (Supplementary Figure 4), TRPV1- (data not shown) and TRPA1-IR (data not shown) inflammatory cells, supporting the evidence that dermal inflammatory cells may co-express different types of cannabinoid receptors.

## Discussion

### Mast cell identification

Mast cells, the first-line responders to allergen stimulation (58) and cell injury (59), play a pivotal role in the neuroimmune response of the skin, and their number increases in skin affected by AD (60, 61).

Several methods have been used to identify the MCs; metachromatic stain with toluidine blue (and other cationic stains) represent the first, rapid and inexpensive method for labeling MCs (62). However, as recently shown, metachromatic stains reveal a lower number of MCs when compared with immunostaining for tryptase (63, 64), which represents the most abundant secretory granule-derived serine proteinase contained in MCs. Thus, immunohistochemistry is presently the best technique for revealing the MCs in tissues, also in dogs (43, 63, 65). The mouse anti-tryptase antibody (clone 3H2643) also immunolabelled some other cells which were not identified with the rabbit anti-tryptase antibody. These cells could have been basophils which could express a lesser amount of tryptase in their cytoplasmic granules (66). However, the expression of the cannabinoid receptors on basophils may be relevant due to the role played by basophils in the Th2 immune response (67).

### Cannabinoid receptors in mast cells

The expression of cannabinoid and cannabinoid-related receptors has already been shown in the MCs of humans (64, 68–70), rodents (13, 71), dogs (43, 44), and cats (50), and in the MC cell line (72).

In the present study, MCs expressed CB2R, GPR55, TRPV1, and TRPA1. The observation of CB2R-IR in the MCs of canine skin is consistent with that of Galiazzo et al. (43) who described CB2R-IR in the MCs of the dog intestine, and Campora et al. (73), who identified CB2R-IR in cells of dog skin showing an MCs-like morphology. Functional studies regarding the MC cell line (RBL2H3) showed that the CB2R modulates MC degranulation and suppresses their proinflammatory response (74), and that the activation of the CB2R on MCs reduces the release of peripheral mediators of nociceptors, such as the nerve growth factor (NGF), serotonin, histamine, and cytokines (75). An interesting study on mice showed that AEA inhibits MC degranulation by means of a mechanism which includes the participation of the CB2R and GPR55 which act in close cross-talk (76).

The observation of GPR55-IR in the MCs of dog skin, which is consistent with data obtained in the dog intestine (43), is promising evidence since the activation of this receptor seems to have anti-inflammatory effects by inhibiting the MC-mediated release of the NGF, as observed in cultures of human MCs (77). In dogs, it has also been shown that the NGF is one of the mediators of the pruritogenic pathways (8). Sensory nerves within the skin, under stressful conditions, can release neuropeptides, such as substance P (SP), the calcitonin gene-related peptide and the vasoactive intestinal peptide, which may activate a neuroimmune response by acting on the MCs located in close proximity to the sensory nerves (78, 79). On the other hand, upon activation, MCs may release the NGF and other neurotrophins, and other powerful mediators, such as histamine, tryptase, and cytokines, which can contribute to hyperinnervation and angiogenesis (77), and stimulate the respective receptors on itch-mediating sensory nerves (12, 80). Therefore, modulation of the release of the NGF by GPR55, although not yet demonstrated in dogs, can lead us to speculate that this receptor, in addition to its antinociceptive properties, may also exert an antipruritic effect (81).

The transient receptor potential vanilloid 1 is preferentially expressed in the sensory neurons of the peripheral nervous system in which it is primarily expressed by the nociceptor neurons of the dorsal root ganglia (DRG) (49). However, TRPV1 seems to also be involved in itching conditions, such as AD (82). The expression of TRPV1-IR in the MCs of dogs is consistent with the expression of the same receptor in the MCs of rodents (71) and humans (77, 78). By using a mouse strain (Nc/Nga) commonly utilized as a model for AD studies, it has been shown that the antagonism of TRPV1 attenuates the itching symptoms induced by house-dust mite allergens (83). In addition, it has been demonstrated that the activation of TRPV1 reduces itching in humans (84) and dogs (85).

The TRPA1 is expressed in neuronal tissues, especially in primary sensory neurons, in which it mediates the peripheral and central processing of pain, itching, and thermal (cold) sensations. Sensory neurons expressing TRPA1-IR usually co-express TRPV1-IR (86). However, TRPA1 is also functionally expressed in skin cells, such as keratinocytes, MCs, macrophages, DCs, melanocytes, and endothelial cells (45, 87–89). The observation of TRPA1-IR in the MCs of the AD-dogs was consistent with the findings of Oh et al. (87) who showed TRPA1 in the MCs of AD lesions of humans and mice. The Authors also showed that IL-13, one of the increasing cytochines in the lesional atopic skin of dogs (90), represents a potent stimulator of TRPA1 expression in MCs and sensory neurons (87). In addition, Kang et al. (91) showed that, in mice, the blockade of TRPA1 inhibits MC degranulation and the production of Th2 cytokine IL-13. More recently, it has been shown that, in a murine model of experimentally induced AD, the genetic deletion of TRPA1 attenuated the pathological findings of AD, including dermal infiltration by MCs and macrophages, Th2 cytokines, and pruritus (92). All these findings robustly emphasize the potential advantage of TRPA1 antagonists as therapy for pathological itching conditions in dogs, although no preclinical or clinical studies have previously been carried out on the skin of this species.

### Cannabinoid and cannabinoid-related receptors in macrophages

Macrophages are critically important in the AD pathogenesis, and they are one of the major components of the dermal infiltrate in chronic lesions of human (21, 93) and canine AD (6, 61, 94). Macrophages interact with lymphocytes to start the acquired immune response, acting as antigen presenting cells and releasing cytokines (95). Coordinated interaction between macrophages, and T and B cells is required to obtain a good immune response. It has been shown that MCs may recruit macrophages by means of the release of proinflammatory mediators (96). It is plausible to consider that an alteration to any one or all of these cell types could reduce the effectiveness of the immune system.

Macrophages of the AD-dogs showed CB2R-IR, a finding consistent with a previous study on the human intestine (97) and mouse skin (98). In mice and humans, the CB2R was shown to modulate macrophages in response to chemoattractants (98) and to switch the polarization of M1 macrophages into M2 macrophages (99, 100).

Macrophages of the skin of the AD-dogs showed GPR55-IR, a finding consistent with those obtained in the macrophages of rodents, humans (101, 102) and dogs (43). Macrophages of the skin of the AD-dogs also expressed TRPV1-IR; this finding supported a recent study which showed the expression of TRPV1 on both the mRNA and the protein levels in canine peripheral blood mononuclear cells and indicated that this ion channel was functional (103). In addition, there is a study on mice supporting the role of the TRPV1 channel in macrophage activation and the effectiveness of a subset of TRPV1 channel antagonists in suppressing inflammatory responses (104).

Large proportions of the dermal macrophages of the AD-dogs showed TRPA1-IR; this evidence was consistent with the data provided in the buccal samples of human patients with oral lichen planus (105) and in skin samples of IL-13–induced chronic AD in mice (87). A recent study involving mice showed that TRPA1 plays a crucial role during AD pathogenesis, and that this receptor could potentially be used as a target for treating chronic skin inflammatory diseases (92); the authors showed that, in addition to lower dermal MC infiltration and proinflammatory cytokines, a lesser infiltration of macrophages was also observed in TRPA1^−/−^ mice (compared to the wild type mice).

### Cannabinoid and cannabinoid-related receptors in dendritic cells

Dendritic cells, which are the most potent antigen-presenting cells of the immune system, are important players during AD pathogenesis (29, 106, 107). It was shown that in the skin of AD-dogs there were significantly more DCs as compared to control skin (107, 108).

The dendritic cells of the AD-dogs, although not differentiable from macrophages (since both cell types express IBA1 in dog skin) (51), showed immunoreactivity for all the receptors studied. Although purely speculative (at present, there are no functional data regarding dogs), it can be hypothesized that, even in dogs, DCs might be potential targets for cannabinoid-mediated modulation. The expression of cannabinoid receptors on DCs has already been reported by authors who showed that the ECS could regulate DC growth, maturation, and their antigen presenting and T cell stimulatory capacities (6, 109–111). Studies involving humans have shown that the stimulation of DCs with CB2R agonists reduced their cytokine production (109). In mice it has been shown that AEA may induce DC apoptosis by engaging the CB1R and the CB2R (110), and that CB2R signaling may affect DC migration primarily by means of the inhibition of matrix metalloproteinase 9 expression (112).

This is the first report regarding the expression of GPR55-IR in the DCs of dogs; this finding is consistent with those obtained involving human and mouse DCs (113, 114). At present, the regulatory mechanism of GPR55 within the DC population is poorly understood.

The dendritic cells of the AD-dogs expressed TRPV1-IR, as shown in human DCs (115). The role of this channel in the innate immunity process has been shown in mice, in which capsaicin (TRPV1 agonist) promoted maturation and the migration of skin DCs to draining lymph nodes (116).

The TRPA1 was also largely expressed by the DCs of the AD-dogs. It appears to be the first evidence of immunoreactivity of these receptors on DCs since, in the literature, there is little and controversial evidence regarding the expression of the TRPA1 on human DCs (117, 118).

### Cannabinoid receptors in T lymphocytes

Atopic dermatitis-related hypersensitivity is considered to be a Th2-polarized lymphocyte response in which a large number of Th2 genes are upregulated (1, 8, 18, 119). The immune responses of Th2 lymphocytes may drive in the s.c. extrinsic AD (3) allergen-specific IgE production (120, 121), and encourage the development of MCs and eosinophils (122). In addition, the transcriptional effect of Th2 type cytokines seems to reduce the production of filaggrin by keratinocytes, and alter the skin barrier function (123).

The expression of the CB2R-, GPR55-, and TRPA1-IR by *T*-cells indicates that a therapeutic effect of cannabinoid molecules in dogs with AD may also be at the level of T cell trafficking. There are studies which report that CBD suppresses T cell function and that palmitoylethanolamide (PEA) could directly inhibit T-cell responses by reducing their production of lymphokines (124–126). In the current study, only a very small proportion of *T*-cells expressed TRPV1-IR.

### Cannabinoid receptors in calprotectin immunoreactive neutrophils

Neutrophils, together with eosinophils and B-lymphocytes, represent small subpopulations of skin-infiltrating cells of canine AD (122, 127). However, there are findings obtained in mice showing that neutrophils play a key role in chronic itch and inflammation (128). Neutrophils, which may be recruited in the skin infiltrate of AD-dogs by interleukin-17 (IL-17), produced by CD4+ Th cells, which control neutrophil homeostasis, seem to play an important role in the development of the Th2 response (129). It has been shown that, in canine AD, there is an increase in Th17 lymphocytes (119, 130). However, a recent study has evaluated the mRNA and protein expression of IL-17 and its receptor in the skin of healthy and atopic dogs, and showed that there was no significant difference in the expression of IL-17 and its receptor between healthy and atopic skin (131).

A large percentage of CAL immunoreactive neutrophils co-expressed GPR55-IR; this finding was consistent with the evidence of GPR55 in human neutrophils in which its activation inhibits their degranulation and the release of reactive oxygen species (132). As shown in human neutrophils (118, 133), also in dogs (current study), no CAL immunoreactive cells expressed TRPA1-IR. Moreover, TRPV1-IR was not expressed by CAL positive neutrophils in the AD-dogs.

### The role of endocannabinoids and phytocannabinoids in atopic dermatitis

Considering the general up-regulation of endocannabinoids (134), and cannabinoid and cannabinoid-related receptors in AD lesions of humans (135) and animals (45, 73), it is reasonable to consider the hypothetical role played by endocannabinoid molecules as well as non-psychotropic *Cannabis* derivates, such as CBD, cannabigerol (CBG) and cannabichromene (CBC), in counteracting the inflammation and itching when AD is present.

In recent years, CBD has garnered significant attention owing to its therapeutic potential in skin disorders (136, 137). The functional activity of CBD on the CB1R is low whereas, on the CB2R, it acts as a weak agonist. Cannabidiol acts as an “indirect” CB1R/CB2R agonist by inhibiting the enzymatic hydrolysis of AEA (138). Cannabichromene may also contribute to the potential therapeutic effectiveness of some *Cannabis* preparations by means of the CB2R-mediated modulation of inflammation (139). The antagonistic effect of CBD on GPR55 seems to prevent inflammation and neuropathic pain by causing the overexpression of endocannabinoids and IL-10 (140). Cannabidiol acts as an agonist and desensitizer of the TRPV1 channel, leading to analgesic and anti-inflammatory effects and to the relief of itching (141). In the same way, CBG activates TRPV1 and inhibits the reuptake of endocannabinoids (142).

In rats, CBD is a potent TRPA1 agonist and desensitizer (40, 141). Other *Cannabis* components, such as THC, CBC and CBG, may activate TRPA1 (142, 143).

The endocannabinoid PEA, which reduces MCs degranulation (13) by means of its interaction with CB2R, also represents a promising molecule to contrast the inflammation and itching in dogs with AD, as has been shown in mice (144). In dogs, it has been shown that the topical application of PEA reduces MC degranulation, and histamine-induced itching and vasodilatation (145). In a rat model of MC cell lines, it has been shown that PEA, which acts as a GPR55 agonist (39), controls the MC degranulation and SP-induced histamine release (146). In addition, PEA, as well as CBD and AEA, acts as a TRPV1 agonist and can desensitize the TRPV1 channel (147, 148).

Considering the endocannabinoid and cannabinoid properties, their mechanisms of action and favorable beneficial results in treating other complex diseases, it is believed that they could exert a positive therapeutic effect on some conditions which are still challenging for veterinarians, such as AD, by reducing the associated inflammation and itch, as has also been shown by two recent studies (149, 150).

The results of the present study could additionally support the preclinical and clinical trials regarding those molecules which are active on the skin inflammatory infiltrate characteristic of canine AD and which are, therefore, capable of mitigating the symptoms of this dermatologic disease.

### Limitation

The mRNA and the molecular expression (Wb) of the CB2R, GPR55, TRPV1 and TRPA1 were not considered. Only the expression of cannabinoid and cannabinoid-related receptors in the inflammatory cells of the skin of AD-dogs having lesions was considered whereas neither the skin of AD-dogs not having lesions, nor the normal skin (control dogs), and nor the skin of dogs with non-allergic dermatosis were evaluated. Although many reports have indicated that cannabinoid and cannabinoid-related receptors are overexpressed during skin inflammation, in the present study, the upregulation (or downregulation) of the immunoreactivity of the receptors studied regarding the inflammatory cells of AD dogs was not evaluated. Therefore, to enhance knoweledge regarding the expression and the role of these receptors in canine AD, other routine investigations (molecular and functional) are necessary.

Given the broad expression of the receptors studied in different organs and cell types, which encompasses different biological functions, the development of the *Cannabis*-related drugs should proceed with caution. It must also be taken into account that there can be a great difference between the histological and the functional findings as well as between the results obtained in rodents and those obtained in dogs, or between studies *in vitro* and *in vivo*. Other basic studies are required to support the preclinical and clinical studies regarding the therapeutic use of cannabinoids in dogs. Nevertheless, since the present evidence showed that the receptors identified in MCs, *T*-cells, macrophages, DCs and neutrophils are crucially involved in the pathogenesis of AD, the pharmacological modulation of these channels could be a valuable complementary strategy for local control of the skin inflammation and pruritus observed in AD.

## Conclusion

The evidence regarding the effect of cannabinoid and cannabinoid-related receptors on MCs, macrophages and DCs (CB2R, GPR55, TRPV1, TRPA1), *T*-cells (CB2R, GPR55, TRPA1), and on neutrophils (GPR55) suggests the possibility that the manipulation of the inflammatory cell functions with endocannabinoids and cannabinoids could result in a novel approach to the treatment of AD. Phytocannabinoids could potentially modulate inflammatory responses by regulating more than one underlying mechanism (inflammatory cells, keratinocytes, sensory nerves, fibroblasts, etc.).

## Data availability statement

The raw data supporting the conclusions of this article will be made available by the authors, without undue reservation.

## Ethics statement

All procedures were approved by the National Health Authority (No. 1303/2021) in accordance with DL 26/2014 and European Union Directive 2010/63/EU, under the supervision of the Central Veterinary Service of the University of Bologna. Written informed consent was obtained from the owners for the participation of their animals in this study.

## Author contributions

RC, MM, and GS contributed to the study design. FA provided skin biopsies for the cryosections. FG, MD, RC, CT, and GG were carried out the immunohistochemical experiments. RC and FG: acquisition of data. RC and MM: drafting of the manuscript. All authors interpreted the data, contributed to the study execution, and approved the final manuscript.

## Funding

This study received a grant from ElleVet Sciences (2021).

## Conflict of interest

The authors declare that the research was conducted in the absence of any commercial or financial relationships that could be construed as a potential conflict of interest.

## Publisher's note

All claims expressed in this article are solely those of the authors and do not necessarily represent those of their affiliated organizations, or those of the publisher, the editors and the reviewers. Any product that may be evaluated in this article, or claim that may be made by its manufacturer, is not guaranteed or endorsed by the publisher.

## References

[1] Massimini M Dalle Vedove E Bachetti B Di Pierro F Ribecco C D'Addario C. Polyphenols and cannabidiol modulate transcriptional regulation of Th1/Th2 inflammatory genes related to canine atopic dermatitis. Front Vet Sci. (2021) 8:606197. 10.3389/fvets.2021.606197 33763461 PMC7982812

[2] Halliwell R. Revised nomenclature for veterinary allergy. Vet Immunol Immunopathol. (2006) 114:207–8. 10.1016/j.vetimm.2006.08.013 17005257

[3] Tizard I. Veterinary immunology 10e. London: Elsevier (2018).

[4] Santoro D. Therapies in canine atopic dermatitis: an update. Vet Clin North Am Small Anim Pract. (2019) 49:9–26. 10.1016/j.cvsm.2018.08.002 30262146

[5] De Benedetto A Agnihothri R McGirt LY Bankova LG Beck LA. Atopic dermatitis: a disease caused by innate immune defects? J Invest Dermatol. (2009) 129:14–30. 10.1038/jid.2008.259 19078985

[6] Pucheu-Haston CM Santoro D Bizikova P Eisenschenk MN Marsella R Nuttall T. Review: innate immunity, lipid metabolism and nutrition in canine atopic dermatitis. Vet Dermatol. (2015) 26:104–e28. 10.1111/vde.12199 25728538

[7] Schlotter YM Riemers FM Rutten VP Knol EF Willemse T. Enzymes involved in the conversion of arachidonic acid to eicosanoids in the skin of atopic dogs. Exp Dermatol. (2010) 19:e317–9. 10.1111/j.1600-0625.2009.01037.x 20201960

[8] Olivry T Mayhew D Paps JS Linder KE Peredo C Rajpal D. Early activation of Th2/Th22 inflammatory and pruritogenic pathways in acute canine atopic dermatitis skin lesions. J Invest Dermatol. (2016) 136:1961–9. 10.1016/j.jid.2016.05.117 27342734

[9] Laprais A Dunston SM Torres SMF Favrot C Olivry T. Evaluation of intraepidermal nerve fibers in the skin of normal and atopic dogs. Vet Dermatol. (2017) 28:355–e80. 10.1111/vde.12420 28133844

[10] Mollanazar NK Smith PK Yosipovitch G. Mediators of chronic pruritus in atopic dermatitis: getting the itch out? Clin Rev Allergy Immunol. (2016) 51:263–92. 10.1007/s12016-015-8488-5 25931325

[11] Church MK Kolkhir P Metz M Maurer M. The role and relevance of mast cells in urticaria. Immunol Rev. (2018) 282:232–47. 10.1111/imr.12632 29431202

[12] Siiskonen H Harvima I. Mast cells and sensory nerves contribute to neurogenic inflammation and pruritus in chronic skin inflammation. Front Cell Neurosci. (2019) 13:422. 10.3389/fncel.2019.00422 31619965 PMC6759746

[13] Facci L Dal Toso R Romanello S Buriani A Skaper SD Leon A. Mast cells express a peripheral cannabinoid receptor with differential sensitivity to anandamide and palmitoylethanolamide. Proc Natl Acad Sci USA. (1995) 92:3376–80. 10.1073/pnas.92.8.3376 7724569 PMC42169

[14] De Filippis D D'Amico A Iuvone T. Cannabinomimetic control of mast cell mediator release: new perspective in chronic inflammation. J Neuroendocrinol. (2008) 1:20–5. 10.1111/j.1365-2826.2008.01674.x 18426495

[15] Amin K. The role of mast cells in allergic inflammation. Respir Med. (2012) 106:9–14. 10.1016/j.rmed.2011.09.007 22112783

[16] Esche C de Benedetto A Beck LA. Keratinocytes in atopic dermatitis: inflammatory signals. Curr Allergy Asthma Rep. (2004) 4:276–84. 10.1007/s11882-004-0071-8 15175141

[17] Asahina R Maeda S. A review of the roles of keratinocyte-derived cytokines and chemokines in the pathogenesis of atopic dermatitis in humans and dogs. Vet Dermatol. (2017) 28:16–e5. 10.1111/vde.12351 27426268

[18] Brunner PM Guttman-Yassky E Leung DYM. The immunology of atopic dermatitis and its reversibility with broad-spectrum and targeted therapies. J Allergy Clin Immunol. (2017) 139:S65–76. 10.1016/j.jaci.2017.01.011 28390479 PMC5405702

[19] Han H Roan F Ziegler SF. The atopic march: current insights into skin barrier dysfunction and epithelial cell-derived cytokines. Immunol Rev. (2017) 278:116–30. 10.1111/imr.12546 28658558 PMC5492959

[20] Tey HL Yosipovitch G. Targeted treatment of pruritus: a look into the future. Br J Dermatol. (2011) 165:5–17. 10.1111/j.1365-2133.2011.10217.x 21219293 PMC3125418

[21] Kasraie S Niebuhr M Werfel T. Interleukin (IL)-31 activates signal transducer and activator of transcription (STAT)-1, STAT-5 and extracellular signal-regulated kinase 1/2 and down-regulates IL-12p40 production in activated human macrophages. Allergy. (2013) 68:739–47. 10.1111/all.12152 23621408

[22] McCandless EE Rugg CA Fici GJ Messamore JE Aleo MM Gonzales AJ. Allergen-induced production of IL-31 by canine Th2 cells and identification of immune, skin, and neuronal target cells. Vet Immunol Immunopathol. (2014) 157:42–8. 10.1016/j.vetimm.2013.10.017 24321252

[23] Cevikbas F Wang X Akiyama T Kempkes C Savinko T Antal A. A sensory neuron-expressed IL-31 receptor mediates T helper cell-dependent itch: Involvement of TRPV1 and TRPA1. J Allergy Clin Immunol. (2014) 133:448–60. 10.1016/j.jaci.2013.10.048 24373353 PMC3960328

[24] Nakashima C Otsuka A Kabashima K. Interleukin-31 and interleukin-31 receptor: new therapeutic targets for atopic dermatitis. Exp Dermatol. (2018) 27:327–31. 10.1111/exd.13533 29524262

[25] Datsi A Steinhoff M Ahmad F Alam M Buddenkotte J. Interleukin-31: The “itchy” cytokine in inflammation and therapy. Allergy. [2021] 76:2982–97. 10.1111/all.14791 33629401

[26] Tamamoto-Mochizuki C Olivry T. IL-31 and IL-31 receptor expression in acute experimental canine atopic dermatitis skin lesions. Vet Dermatol. (2021) 32:631–e169. 10.1111/vde.13034 34796564

[27] Enk AH Katz SI. Early events in the induction phase of contact sensitivity. J Invest Dermatol. (1992) 99:39S−41S. 10.1111/1523-1747.ep12668608 1385542

[28] Parameswaran N Patial S. Tumor necrosis factor-alpha signaling in macrophages. Crit Rev Eukaryot Gene Expr. (2010) 20:87–103. 10.1615/critreveukargeneexpr.v20.i2.10 21133840 PMC3066460

[29] Moore PF. A review of histiocytic diseases of dogs and cats. Vet Pathol. (2014) 51:167–84. 10.1177/0300985813510413 24395976

[30] Iannotti FA Di Marzo V Petrosino S. Endocannabinoids and endocannabinoid-related mediators: targets, metabolism and role in neurological disorders. Prog Lipid Res. (2016) 62:107–128. 10.1016/j.plipres.2016.02.002 26965148

[31] Tòth KF Adàm D Birò T Olàh A. Cannabinoid Signaling in the Skin: Therapeutic Potential of the “C(ut)annabinoid” System. Molecules. (2019) 24:918. 10.3390/molecules24050918 30845666 PMC6429381

[32] Silver RJ. The Endocannabinoid system of animals. Animals. (2019) 9:686. 10.3390/ani9090686 31527410 PMC6770351

[33] Di Marzo V. Targeting the endocannabinoid system: to enhance or reduce? Nat Rev Drug Discov. (2008) 7:438–55. 10.1038/nrd2553 18446159

[34] Kupczyk P Reich A Szepietowski JC. Cannabinoid system in the skin—a possible target for future therapies in dermatology. Exp Dermatol. (2009) 18:669–79. 10.1111/j.1600-0625.2009.00923.x 19664006

[35] Donvito G Nass SR Wilkerson JL Curry ZA Schurman LD Kinsey SG. The endogenous cannabinoid system: a budding source of targets for treating inflammatory and neuropathic pain. Neuropsychopharmacology. (2018) 43:52–79. 10.1038/npp.2017.204 28857069 PMC5719110

[36] Avila C Massick S Kaffenberger BH Kwatra SG Bechtel M. Cannabinoids for the treatment of chronic pruritus: a review. J Am Acad Dermatol. (2020) 82:1205–12. 10.1016/j.jaad.2020.01.036 31987788

[37] Kreitzer FR Stella N. The therapeutic potential of novel cannabinoid receptors. Pharmacol Ther. (2009) 122:83–96. 10.1016/j.pharmthera.2009.01.005 19248809 PMC3685492

[38] Morales P Hurst DP Reggio PH. Molecular targets of the phytocannabinoids: a complex picture. Prog Chem Org Nat Prod. (2017) 103:103–31. 10.1007/978-3-319-45541-9_4 28120232 PMC5345356

[39] Petrosino S Di Marzo V. The pharmacology of palmitoylethanolamide and first data on the therapeutic efficacy of some of its new formulations. Br J Pharmacol. (2017) 174:1349–65. 10.1111/bph.13580 27539936 PMC5429331

[40] Mlost J Bryk M Starowicz K. Cannabidiol for pain treatment: focus on pharmacology and mechanism of action. Int J Mol Sci. (2020) 21:8870. 10.3390/ijms21228870 33238607 PMC7700528

[41] Mechoulam R Peters M Murillo-Rodriguez E Hanus LO. Cannabidiol–recent advances. Chem Biodivers. (2007) 4:1678–92. 10.1002/cbdv.200790147 17712814

[42] Pertwee RG. The diverse CB1 and CB2 receptor pharmacology of three plant cannabinoids: delta9-tetrahydrocannabinol, cannabidiol and delta9-tetrahydrocannabivarin. Br J Pharmacol. (2008) 153:199–215. 10.1038/sj.bjp.0707442 17828291 PMC2219532

[43] Galiazzo G Giancola F Stanzani A Fracassi F Bernardini C Forni M. Localization of cannabinoid receptors CB1, CB2, GPR55, and PPARα in the canine gastrointestinal tract. Histochem Cell Biol. (2018) 150:187–205. 10.1007/s00418-018-1684-7 29882158

[44] Gobbo F Sarli G De Silva M Galiazzo G Chiocchetti R Morini M. A double histochemical/immunohistochemical staining for the identification of canine mast cells in light microscopy. Vet Sci. (2021) 8:229. 10.3390/vetsci8100229 34679059 PMC8537840

[45] Chiocchetti R De Silva M Aspidi F Zamith Cunha R Gobbo F Tagliavia C. Distribution of cannabinoid receptors in keratinocytes of healthy dogs and dogs with atopic dermatitis. Front Vet Sci. (2022) 9:915896. 10.3389/fvets.2022.915896 35873682 PMC9305491

[46] Hill PB Lau P Rybnicek J. Development of an owner-assessed scale to measure the severity of pruritus in dogs. Vet Dermatol. (2007) 18:301–8. 10.1111/j.1365-3164.2007.00616.x 17845617

[47] Olivry T Saridomichelakis M Nuttall T Bensignor E Griffin CE Hill PB International Committe on Allergic Diseases of Animals (ICADA). Validation of the Canine Atopic Dermatitis Extent and Severity Index (CADESI)-4, a simplified severity scale for assessing skin lesions of atopic dermatitis in dogs. Vet Dermatol. (2014) 25:77–85. 10.1111/vde.12107 24461108

[48] Gross TL Ihrke PJ Walder EJ Affolter VK. Skin Diseases of the Dog and Cat. Ames: Blackwell Publishing (2005).

[49] Chiocchetti R Galiazzo G Tagliavia C Stanzani A Giancola F Menchetti M. Cellular distribution of canonical and putative cannabinoid receptors in canine cervical dorsal root ganglia. Front Vet Sci. (2019) 6:313. 10.3389/fvets.2019.00313 31608295 PMC6761858

[50] Stanzani A Galiazzo G Giancola F Tagliavia C De Silva M Pietra M. Localization of cannabinoid and cannabinoid related receptors in the cat gastrointestinal tract. Histochem Cell Biol. (2020) 153:339–56. 10.1007/s00418-020-01854-0 32095931

[51] Pierezan F Mansell J Ambrus A Rodrigues Hoffmann A. Immunohistochemical expression of ionized calcium binding adapter molecule 1 in cutaneous histiocytic proliferative, neoplastic and inflammatory disorders of dogs and cats. J Comp Pathol. (2014) 151:347–51. 10.1016/j.jcpa.2014.07.003 25172051

[52] Villiers E Baines S Law AM Mallows V. Identification of acute myeloid leukemia in dogs using flow cytometry with myeloperoxidase, MAC387, and a canine neutrophil-specific antibody. Vet Clin Pathol. (2006) 35:55–71. 10.1111/j.1939-165x.2006.tb00089.x 16511792

[53] Kerkhoff C Voss A Scholzen TE Averill MM Zänker KS Bornfeldt KE. Novel insights into the role of S100A8/A9 in skin biology. Exp Dermatol. (2012) 21:822–6. 10.1111/j.1600-0625.2012.01571.x 22882537 PMC3498607

[54] Mozos E Pérez J Day MJ Lucena R Ginel PJ. Leishmaniosis and generalized demodicosis in three dogs: a clinicopathological and immunohistochemical study. J Comp Pathol. (1999) 120:257–68. 10.1053/jcpa.1998.0273 10213670

[55] Nolte A Junginger J Baum B Hewicker-Trautwein M. Heterogeneity of macrophages in canine histiocytic ulcerative colitis. Innate Immun. (2017) 23:228–39. 10.1177/1753425916686170 28100085

[56] Dandrieux JR Martinez Lopez LM Stent A Jergens A Allenspach K Nowell CJ. Changes in duodenal CD163-positive cells in dogs with chronic enteropathy after successful treatment. Innate Immun. (2018) 24:400–10. 10.1177/1753425918799865 30223681 PMC6830873

[57] Levi M Parenti F Muscatello LV Battaia S Santilli R Perego M. Pathological findings of canine idiopathic pericarditis and pericardial mesotheliomas: correlation with clinical and survival data. Vet Sci. (2021) 8:162. 10.3390/vetsci8080162 34437484 PMC8402876

[58] Modena BD Kristen D Andrew A. White Emerging concepts: mast cell involvement in allergic diseases. Translat Res. (2016) 174:98–121. 10.1016/j.trsl.2016.02.011 26976119

[59] Enoksson M Lyberg K Moller-Westerberg C Fallon PG Nilsson G Lunderius-Andersson C. Mast cells as sensors of cell injury through IL-33 recognition. J Immunol. (2011) 186:2523–8. 10.4049/jimmunol.1003383 21239713

[60] Soter NA. Morphology of atopic eczema. Allergy. (1989) 9:16–9. 10.1111/j.1398-9995.1989.tb04310.x 2683838

[61] Wilkie JS Yager JA Eyre P Parker WM. Morphometric analyses of the skin of dogs with atopic dermatitis and correlations with cutaneous and plasma histamine and total serum IgE. Vet Pathol. (1990) 27:179–86. 10.1177/030098589002700305 2353419

[62] Ribatti D. The staining of mast cells: a historical overview. Int Arch Allergy Immunol. (2018) 176:55–60. 10.1159/000487538 29597213

[63] He SH. Key role of mast cells and their major secretory products in inflammatory bowel disease. World J Gastroenterol. (2004) 10:309–18. 10.3748/wjg.v10.i3.309 14760748 PMC4724914

[64] Ständer S Moormann C Schumacher M Buddenkotte J Artuc M Shpacovitch V. Expression of vanilloid receptor subtype 1 in cutaneous sensory nerve fibers, mast cells, and epithelial cells of appendage structures. Exp Dermatol. (2004) 13:129–39. 10.1111/j.0906-6705.2004.0178.x 14987252

[65] Atiakshin D Samoilova V Buchwalow I Boecker W Tiemann M. Characterization of mast cell populations using different methods for their identification. Histochem Cell Biol. (2017) 147:683–94. 10.1007/s00418-017-1547-7 28243739

[66] Jogie-Brahim S Min HK Fukuoka Y Xia HZ Schwartz LB. Expression of alpha-tryptase and beta-tryptase by human basophils. J Allergy Clin Immunol. (2004) 113:1086–92. 10.1016/j.jaci.2004.02.032 15208589

[67] Yamanishi Y Miyake K Iki M Tsutsui H Karasuyama H. Recent advances in understanding basophil-mediated Th2 immune responses. Immunol Rev. (2017) 278:237–45. 10.1111/imr.12548 28658549

[68] Lazzeri M Vannucchi MG Zardo C Spinelli M Beneforti P Turini D. mmunohistochemical evidence of vanilloid receptor 1 in normal human urinary bladder. Eur Urol. (2004) 46:792–8. 10.1016/j.eururo.2004.08.007 15548449

[69] Ständer S Schmelz M Metze D Luger T Rukwied R. Distribution of cannabinoid receptor 1 (CB1) and 2 (CB2) on sensory nerve fibers and adnexal structures in human skin. J Dermatol Sci. (2005) 38:177–88. 10.1016/j.jdermsci.2005.01.007 15927811

[70] Rasul A El-Nour H Lonne-Rahm SB Fransson O Johansson C Johansson B. Serotonergic markers in atopic dermatitis. Acta Derm Venereol. (2016) 96:732–6. 10.2340/00015555-2354 26831833

[71] Bíró T Maurer M Modarres S Lewin NE Brodie C Acs G. Characterization of functional vanilloid receptors expressed by mast cells. Blood. (1998) 91:1332–40. 9454764

[72] Samson MT Small-Howard A Shimoda LM Koblan-Huberson M Stokes AJ Turner H. Differential roles of CB1 and CB2 cannabinoid receptors in mast cells. J Immunol. (2003) 170:4953–62. 10.4049/jimmunol.170.10.4953 12734338

[73] Campora L Miragliotta V Ricci E Cristino L Di Marzo V Albanese F. Cannabinoid receptor type 1 and 2 expression in the skin of healthy dogs and dogs with atopic dermatitis. Am J Vet Res. (2012) 73:988–95. 10.2460/ajvr.73.7.988 22738050

[74] Small-Howard AL Shimoda LM Adra CN Turner H. Anti-inflammatory potential of CB1-mediated AMP elevation in mast cells. Biochemical Journal. (2005) 388:465–73. 10.1042/BJ20041682 15669919 PMC1138953

[75] Malan TP Ibrahim MM Vanderah TW Makriyannis A Porreca F. Inhibition of pain responses by activation of CB(2) cannabinoid receptors. Chem Phys Lipids. (2002) 121:191–200. 10.1016/s0009-3084(02)00155-x 12505700

[76] Cruz SL Sánchez-Miranda E Castillo-Arellano JI Cervantes-Villagrana RD Ibarra-Sánchez A González-Espinosa C. Anandamide inhibits FcεRI-dependent degranulation and cytokine synthesis in mast cells through CB2 and GPR55 receptor activation. Possible involvement of CB2-GPR55 heteromers. Int Immunopharmacol. (2018) 64:298–307. 10.1016/j.intimp.2018.09.006 30243065

[77] Cantarella G Scollo M Lempereur L Saccani-Jotti G Basile F Bernardini R. Endocannabinoids inhibit release of nerve growth factor by inflammation-activated mast cells. Biochem Pharmacol. (2011) 82:380–8. 10.1016/j.bcp.2011.05.004 21601562

[78] Siebenhaar F Magerl M Peters EM Hendrix S Metz M Maurer M. Mast cell-driven skin inflammation is impaired in the absence of sensory nerves. J Allergy Clin Immunol. (2008) 121:955–61. 10.1016/j.jaci.2007.11.013 18158175

[79] Serhan N Basso L Sibilano R Petitfils C Meixiong J Bonnart C. House dust mites activate nociceptor-mast cell clusters to drive type 2 skin inflammation. Nat Immunol. (2019) 20:1435–43. 10.1038/s41590-019-0493-z 31591569 PMC6858877

[80] Rossbach K Nassenstein C Gschwandtner M Schnell D Sander K Seifert R. Histamine H1, H3 and H4 receptors are involved in pruritus. Neuroscience. (2011) 190:89–102. 10.1016/j.neuroscience.2011.06.002 21689731

[81] Schlosburg JE Boger DL Cravatt BF Lichtman AH. Endocannabinoid modulation of scratching response in an acute allergenic model: a new prospective neural therapeutic target for pruritus. J Pharmacol Exp Ther. (2009) 329:314–23. 10.1124/jpet.108.150136 19168707 PMC2670585

[82] Sun S Dong X TRP channels and itch. Semin Immunopathol. (2016) 38:293–307. 10.1007/s00281-015-0530-4 26385480 PMC4798920

[83] Yun JW Seo JA Jang WH Koh HJ Bae IH Park YH. Antipruritic effects of TRPV1 antagonist in murine atopic dermatitis and itching models. J Invest Dermatol. (2011) 131:1576–9. 10.1038/jid.2011.87 21471988

[84] Weisshaar E Dunker N Gollnick H. Topical capsaicin therapy in humans with hemodialysis-related pruritus. Neurosci Lett. (2003) 345:192–4. 10.1016/s0304-3940(03)00511-1 12842288

[85] Marsella R Nicklin CF Melloy C. The effects of capsaicin topical therapy in dogs with atopic dermatitis: a randomized, double-blinded, placebo-controlled, cross-over clinical trial. Vet Dermatol. (2002) 13:131–9. 10.1046/j.1365-3164.2002.00292.x 12074702

[86] Kobayashi K Fukuoka T Obata K Yamanaka H Dai Y Tokunaga A. Distinct expression of TRPM8, TRPA1, and TRPV1 mRNAs in rat primary afferent neurons with adelta/c-fibers and colocalization with trk receptors. J Comp Neurol. (2005) 493:596–606. 10.1002/cne.20794 16304633

[87] Oh MH Oh SY Lu J Lou H Myers AC Zhu Z. TRPA1-dependent pruritus in IL-13-induced chronic atopic dermatitis. J Immunol. (2013) 191:5371–82. 10.4049/jimmunol.1300300 24140646 PMC4175413

[88] Moore C Gupta R Jordt SE Chen Y Liedtke WB. Regulation of pain and itch by TRP channels. Neurosci Bull. (2018) 34:120–42. 10.1007/s12264-017-0200-8 29282613 PMC5799130

[89] Maglie R Souza Monteiro de Araujo D Antiga E Geppetti P Nassini R De Logu F. The Role of TRPA1 in skin physiology and pathology. Int J Mol Sci. (2021) 22:3065. 10.3390/ijms22063065 33802836 PMC8002674

[90] Marsella R Olivry T Maeda S. Cellular and cytokine kinetics after epicutaneous allergen challenge (atopy patch testing) with house dust mites in high-IgE beagles. Vet Dermatol. (2006) 17:111–20. 10.1111/j.1365-3164.2006.00508.x 16515653

[91] Kang J Ding Y Li B Liu H Yang X Chen M. TRPA1 mediated aggravation of allergic contact dermatitis induced by DINP and regulated by NF-κB activation. Sci Rep. (2017) 7:43586. 10.1038/srep43586 28240277 PMC5327402

[92] Zeng D Chen C Zhou W Ma X Pu X Zeng Y. TRPA1 deficiency alleviates inflammation of atopic dermatitis by reducing macrophage infiltration. Life Sci. (2021) 266:118906. 10.1016/j.lfs.2020.118906 33338502

[93] Leung DY. Atopic dermatitis: the skin as a window into the pathogenesis of chronic allergic diseases. J Allergy Clin Immunol. (1995) 96:302–18. 10.1016/s0091-6749(95)70049-8 7560632

[94] Ricklin Gutzwiller ME Moulin HR Zurbriggen A Roosje P Summerfield A. Comparative analysis of canine monocyte- and bone-marrow-derived dendritic cells. Vet Res. (2010) 41:40. 10.1051/vetres/2010012 20167201 PMC2839791

[95] Honda T Egawa G Kabashima K. Antigen presentation and adaptive immune responses in skin. Int Immunol. (2019) 31:423–9. 10.1093/intimm/dxz005 30668771

[96] He S Peng Q Walls AF. Potent induction of a neutrophil and eosinophil-rich infiltrate in vivo by human mast cell tryptase: selective enhancement of eosinophil recruitment by histamine. J Immunol. (1997) 159:6216–25. 9550425

[97] Wright K Rooney N Feeney M Tate J Robertson D Welham M. Differential expression of cannabinoid receptors in the human colon: cannabinoids promote epithelial wound healing. Gastroenterology. (2005) 129:437–53. 10.1016/j.gastro.2005.05.026 16083701

[98] Zheng JL Yu TS Li XN Fan YY Ma WX Du Y. Cannabinoid receptor type 2 is time-dependently expressed during skin wound healing in mice. Int J Legal Med. (2012) 126:807–14. 10.1007/s00414-012-0741-3 22814434

[99] Du Y Ren P Wang Q Jiang SK Zhang M Li JY. Cannabinoid 2 receptor attenuates inflammation during skin wound healing by inhibiting M1 macrophages rather than activating M2 macrophages. J Inflamm (Lond). (2018) 15:25. 10.1186/s12950-018-0201-z 30534003 PMC6278147

[100] Tarique AA Evron T Zhang G Tepper MA Morshed MM Andersen ISG. Anti-inflammatory effects of lenabasum, a cannabinoid receptor type 2 agonist, on macrophages from cystic fibrosis. J Cyst Fibros. (2020) 19:823–9. 10.1016/j.jcf.2020.03.015 32387042

[101] Taylor L Christou I Kapellos TS Buchan A Brodermann MH Gianella-Borradori M. Primary macrophage chemotaxis induced by cannabinoid receptor 2 agonists occurs independently of the CB2 receptor. Sci Rep. (2015) 5:10682. 10.1038/srep10682 26033291 PMC4451551

[102] Lanuti M Talamonti E Maccarrone M Chiurchiù V. Correction: activation of GPR55 receptors exacerbates oxLDL-induced lipid accumulation and inflammatory responses, while reducing cholesterol efflux from human macrophages. PLoS One. (2015) 10:e0131850. 10.1371/journal.pone.0131850 25970609 PMC4430319

[103] Bujak JK Kosmala D Majchrzak-Kuligowska K Bednarczyk P. Functional Expression of TRPV1 Ion Channel in the Canine Peripheral Blood Mononuclear Cells. Int J Mol Sci. (2021) 22:3177. 10.3390/ijms22063177 33804707 PMC8003907

[104] Ninomiya Y Tanuma SI Tsukimoto M. Differences in the effects of four TRPV1 channel antagonists on lipopolysaccharide-induced cytokine production and COX-2 expression in murine macrophages. Biochem Biophys Res Commun. (2017) 484:668–74. 10.1016/j.bbrc.2017.01.173 28153725

[105] Kun J Perkecz A Knie L Sétáló G Jr Tornóczki T Pintér E. TRPA1 receptor is upregulated in human oral lichen planus. Oral Dis. (2017) 23:189–98. 10.1111/odi.12593 27718297

[106] Novak N Bieber T. The role of dendritic cell subtypes in the pathophysiology of atopic dermatitis. J Am Acad Dermatol. (2005) 53:S171–176. 10.1016/j.jaad.2005.04.060 16021172

[107] Ricklin ME Roosje P Summerfield A. Characterization of canine dendritic cells in healthy, atopic, and non-allergic inflamed skin. J Clin Immunol. (2010) 30:845–54. 10.1007/s10875-010-9447-9 20676740

[108] Olivry T Moore PF Affolter VK Naydan DK. Langerhans cell hyperplasia and IgE expression in canine atopic dermatitis. Arch Dermatol Res. (1996) 288:579–85. 10.1007/BF02505260 8919040

[109] Matias I Pochard P Orlando P Salzet M Pestel J Di Marzo V. Presence and regulation of the endocannabinoid system in human dendritic cells. Eur J Biochem. (2002) 269:3771–8. 10.1046/j.1432-1033.2002.03078.x 12153574

[110] Do Y McKallip RJ Nagarkatti M Nagarkatti PS. Activation through cannabinoid receptors 1 and 2 on dendritic cells triggers NF-kappaB-dependent apoptosis: novel role for endogenous and exogenous cannabinoids in immunoregulation. J Immunol. (2004) 173:2373–82. 10.4049/jimmunol.173.4.2373 15294950

[111] Svensson M Chen P Hammarfjord O. Dendritic cell regulation by cannabinoid-based drugs. Pharmaceuticals (Basel). (2010) 3:2733–50. 10.3390/ph3082733 27713374 PMC4033947

[112] Adhikary S Kocieda VP Yen JH Tuma RF Ganea D. Signaling through cannabinoid receptor 2 suppresses murine dendritic cell migration by inhibiting matrix metalloproteinase 9 expression. Blood. (2012) 120:3741–9. 10.1182/blood-2012-06-435362 22972984 PMC3488886

[113] Castillo-Chabeco B Figueroa G Parira T Napuri J Agudelo M. Ethanol-induced modulation of GPR55 expression in human monocyte-derived dendritic cells is accompanied by H4K12 acetylation. Alcohol. (2018) 71:25–31. 10.1016/j.alcohol.2018.05.008 29957399

[114] Tanikawa T Oka S Nakajima K Hayashi Y Nemoto-Sasaki Y Arata Y. Expression and Distribution of GPR55, a Receptor for Lysophosphatidylinositol, in Mouse Tissues and Cells. BPB Reports. (2022) 5:15–20. 10.1248/bpbreports.5.2_16

[115] Tóth BI Benko S Szöllosi AG Kovács L Rajnavölgyi E Bíró T. Transient receptor potential vanilloid-1 signaling inhibits differentiation and activation of human dendritic cells. FEBS Lett. (2009) 583:1619–24. 10.1016/j.febslet.2009.04.031 19397909

[116] Basu S Srivastava P. Immunological role of neuronal receptor vanilloid receptor 1 expressed on dendritic cells. Proc Natl Acad Sci U S A. (2005) 102:5120–5. 10.1073/pnas.0407780102 15793000 PMC555601

[117] Szöllösi AG Oláh A Tóth IB Papp F Czifra G Panyi G. Transient receptor potential vanilloid-2 mediates the effects of transient heat shock on endocytosis of human monocyte-derived dendritic cells. FEBS Lett. (2013) 587:1440–5. 10.1016/j.febslet.2013.03.027 23542034

[118] Naert R López-Requena A Talavera K. TRPA1 Expression and Pathophysiology in Immune Cells. Int J Mol Sci. (2021) 22:11460. 10.3390/ijms222111460 34768891 PMC8583806

[119] Marsella R. Advances in our understanding of canine atopic dermatitis. Vet Dermatol. (2021) 21:1265. 10.1111/vde.12965 33891338

[120] Majewska A Gajewska M Dembele K Maciejewski H Prostek A Jank M. Lymphocytic, cytokine and transcriptomic profiles in peripheral blood of dogs with atopic dermatitis. BMC Vet Res. (2016) 12:174. 10.1186/s12917-016-0805-6 27553600 PMC4995625

[121] Vogelnest L. Canine atopic dermatitis: a common, chronic and challenging dermatosis. Vet Rec. (2021) 188:185–7. 10.1002/vetr.273 33666979

[122] Hill PB Olivry T. The ACVD task force on canine atopic dermatitis (V): biology and role of inflammatory cells in cutaneous allergic reactions. Vet Immunol Immunopathol. (2001) 81:187–98. 10.1016/s0165-2427(01)00310-5 11553379

[123] Combarros D Cadiergues MC Simon M. Update on canine filaggrin: a review. Vet Q. (2020) 40:162–8. 10.1080/01652176.2020.1758357 32308144 PMC7241532

[124] Wu HY Chu RM Wang CC Lee CY Lin SH Jan TR. Cannabidiol-induced apoptosis in primary lymphocytes is associated with oxidative stress-dependent activation of caspase-8. Toxicol Appl Pharmacol. (2008) 226:260–70. 10.1016/j.taap.2007.09.012 17950393

[125] Kaplan BLF Springs AEB Kaminski NE. The profile of immune modulation by cannabidiol (CBD) involves deregulation of nuclear factor of activated T cells (NFAT). Biochem Pharmacol. (2008) 76:726–37. 10.1016/j.bcp.2008.06.022 18656454 PMC2748879

[126] Chiurchiù V van der Stelt M Centonze D Maccarrone M. The endocannabinoid system and its therapeutic exploitation in multiple sclerosis: clues for other neuroinflammatory diseases. Prog Neurobiol. (2018) 160:82–100. 10.1016/j.pneurobio.2017.10.007 29097192

[127] Olivry T Naydan DK Moore PF. Characterization of the cutaneous inflammatory infiltrate in canine atopic dermatitis. Am J Dermatopathol. (1997) 19:477–86. 10.1097/00000372-199710000-00008 9335242

[128] Walsh CM Hill RZ Schwendinger-Schreck J Deguine J Brock EC Kucirek N. Neutrophils promote CXCR3-dependent itch in the development of atopic dermatitis. Elife. (2019) 8:e48448. 10.7554/eLife.48448 31631836 PMC6884397

[129] Asahina R Kamishina H Kamishina H Maeda S. Gene transcription of pro-inflammatory cytokines and chemokines induced by IL-17A in canine keratinocytes. Vet Dermatol. (2015) 26:426–31. 10.1111/vde.12244 26248589

[130] Jassies-van der Lee A Rutten VP Bruijn J Willemse T Broere F. CD4+ and CD8+ skin-associated T lymphocytes in canine atopic dermatitis produce interleukin-13, interleukin-22 and interferon-γ and contain a CD25+ FoxP3+ subset. Vet Dermatol. (2014) 25:456–e72. 10.1111/vde.12140 24913127

[131] Shiomitsu S Gillen J Frasca S Jr Santoro D. Evaluation of the cutaneous expression of IL-17, IL-22, IL-31, and their receptors in canine atopic dermatitis. Res Vet Sci. (2021) 136:74–80. 10.1016/j.rvsc.2020.12.015 33588097

[132] Balenga NA Aflaki E Kargl J Platzer W Schröder R Blättermann S. GPR55 regulates cannabinoid 2 receptor-mediated responses in human neutrophils. Cell Res. (2011) 21:1452–69. 10.1038/cr.2011.60 21467997 PMC3132458

[133] Foote JR Behe P Frampton M Levine AP Segal AW. An exploration of charge compensating ion channels across the phagocytic vacuole of neutrophils. Front Pharmacol. (2017) 8:94. 10.3389/fphar.2017.00094 28293191 PMC5329019

[134] Abramo F Campora L Albanese F. della Valle MF, Cristino L, Petrosino S, et al. Increased levels of palmitoylethanolamide and other bioactive lipid mediators and enhanced local mast cell proliferation in canine atopic dermatitis. BMC Vet Res. (2014) 10:21. 10.1186/1746-6148-10-21 24423192 PMC3923739

[135] Martín-Fontecha M Eiwegger T Jartti T Rueda-Zubiaurre A Tiringer K Stepanow J. The expression of cannabinoid receptor 1 is significantly increased in atopic patients. J Allergy Clin Immunol. (2014) 133:926-9.e2. 10.1016/j.jaci.2013.12.1032 24486070

[136] Petrosino S Verde R Vaia M Allarà M Iuvone T Di Marzo V. Anti- inflammatory properties of cannabidiol, a nonpsychotropic cannabinoid, in experimental allergic contact dermatitis. J Pharmacol Exp Ther. (2018) 365:652–63. 10.1124/jpet.117.244368 29632236

[137] Baswan SM Klosner AE Glynn K Rajgopal A Malik K Yim S. Therapeutic potential of cannabidiol (CBD) for skin health and disorders. Clin Cosmet Investig Dermatol. (2020) 13:927–42. 10.2147/CCID.S286411 33335413 PMC7736837

[138] Ligresti A De Petrocellis L Di Marzo V. From phytocannabinoids to cannabinoid receptors and endocannabinoids: pleiotropic physiological and pathological roles through complex pharmacology. Physiol Rev. (2016) 96:1593–659. 10.1152/physrev.00002.2016 27630175

[139] Udoh M Santiago M Devenish S McGregor IS Connor M. Cannabichromene is a cannabinoid CB(2) receptor agonist. Br J Pharmacol. (2019) 176:4537–47. 10.1111/bph.14815 31368508 PMC6932936

[140] Sunda F Arowolo A. A molecular basis for the anti-inflammatory and anti-fibrosis properties of cannabidiol. FASEB J. (2020) 34:14083–92. 10.1096/fj.202000975R 32885502

[141] De Petrocellis L Ligresti A Schiano Moriello A Allara M Bisogno T Petrosino S. Effects of cannabinoids and cannabinoid-enriched Cannabis extracts on TRP channels and endocannabinoid metabolic enzymes. Br J Pharmacol. (2011) 163:1479–94. 10.1111/j.1476-5381.2010.01166.x 21175579 PMC3165957

[142] Borrelli F Pagano E Romano B Panzera S Maiello F Coppola D. Colon carcinogenesis is inhibited by the TRPM8 antagonist cannabigerol, a Cannabis-derived non-psychotropic cannabinoid. Carcinogenesis. (2014) 35:2787–97. 10.1093/carcin/bgu205 25269802

[143] De Petrocellis L Vellani V Schiano-Moriello A Marini P Magherini PC Orlando P. Plant-derived cannabinoids modulate the activity of transient receptor potential channels of ankyrin type-1 and melastatin type-8. J Pharmacol Exp Ther. (2008) 325:1007–15. 10.1124/jpet.107.134809 18354058

[144] Vaia M Petrosino S De Filippis D Negro L Guarino A Carnuccio R. Palmitoylethanolamide reduces inflammation and itch in a mouse model of contact allergic dermatitis. Eur J Pharmacol. (2016) 791:669–74. 10.1016/j.ejphar.2016.10.005 27720681

[145] Cerrato S Brazis P della Valle MF Miolo A Puigdemont A. Effects of palmitoylethanolamide on immunologically induced histamine, PGD2 and TNFalpha release from canine skin mast cells. Vet Immunol Immunopathol. (2010) 133:9–15. 10.1016/j.vetimm.2009.06.011 19625089

[146] Petrosino S Schiano Moriello A Verde R Allarà M Imperatore R Ligresti A. Palmitoylethanolamide counteracts substance P-induced mast cell activation in vitro by stimulating diacylglycerol lipase activity. J Neuroinflammation. (2019) 16:274. 10.1186/s12974-019-1671-5 31878942 PMC6933707

[147] Di Marzo V De Petrocellis L Fezza F Ligresti A Bisogno T. Anandamide receptors. Prostaglandins Leukot Essent Fatty Acids. (2002) 66:377–91. 10.1054/plef.2001.0349 12052051

[148] Ross RA. Anandamide and vanilloid TRPV1 receptors. Br J Pharmacol. (2003) 140:790–801. 10.1038/sj.bjp.0705467 14517174 PMC1574087

[149] CannPal Animal Therapeutics Limited. CBD Substantially Improves Atopic Dermatitis Symptoms in Dogs. Available online at: https://www.prnewswire.com/news-releases/cbd-substantially-improves-atopic-dermatitis-symptoms-in-dogs-301095965.html. (accessed July 21, 2020).

[150] ElleVet Sciences. ElleVet Sciences Announce Results of Atopic Dermatitis Study Using Its CBD+CBDA Oil on Dogs. Available online at: https://www.prnewswire.com/news-releases/ellevet-sciences-announces-results-of-atopic-dermatitis-study-using-its-cbdcbda-oil-on-dogs-301189648.html. (accessed July 9, 2020).

