# Supplementary material for: Cannabinoid receptors in the inflammatory cells of canine atopic dermatitis
Source: Front Vet Sci. 2022 Sep 15;9:987132. doi: 10.3389/fvets.2022.987132 (PMC9521433; doi:10.3389/fvets.2022.987132)
Supplement: Supplementary file 1 [file Data_Sheet_1.PDF]

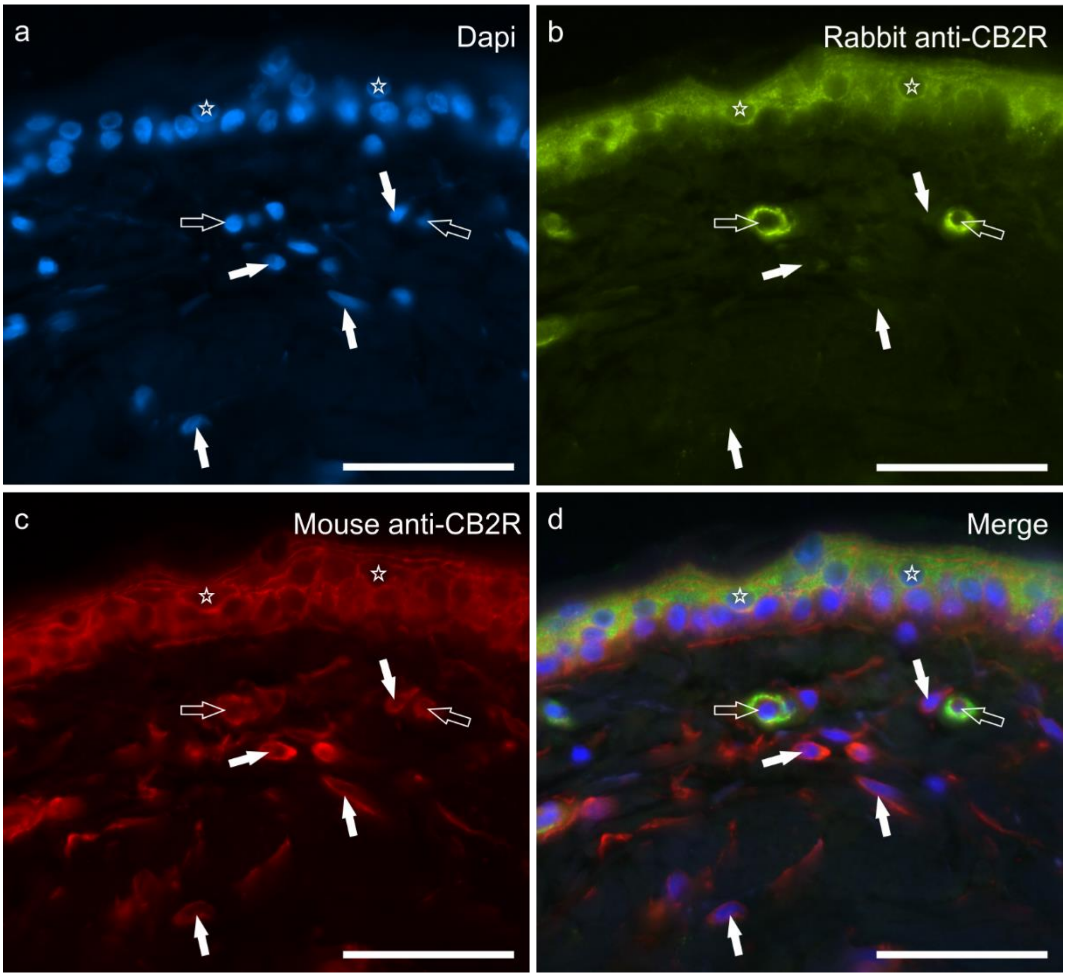

Supplementary Fig. 1.

Photomicrographs of a cryosection of the skin of a dog with atopic dermatitis on which two different antibodies (one raised in rabbit [ab45942] and the other one in mouse [sc-293188]) against cannabinoid receptor 2 (CB2R) were applied. Although both the anti-CB2R antibodies identified the same cellular elements in the dog skin, the immunolabeling of the dermal inflammatory cells was more evident with the mouse anti-CB2R antibody (white arrows), whereas the immunolabeling of the vascular endothelial was more evident with the use of the rabbit anti-CB2R (open arrows). Stars indicate two Dapi-labelled nuclei of the keratinocytes which showed bright CB2R immunolabeling with both the anti-CB2R antibodies.

Bar: 50 µm.
