# Supplementary material for: Cannabinoid receptors in the inflammatory cells of canine atopic dermatitis
Source: Front Vet Sci. 2022 Sep 15;9:987132. doi: 10.3389/fvets.2022.987132 (PMC9521433; doi:10.3389/fvets.2022.987132)
Supplement: Supplementary file 2 [file Data_Sheet_2.PDF]

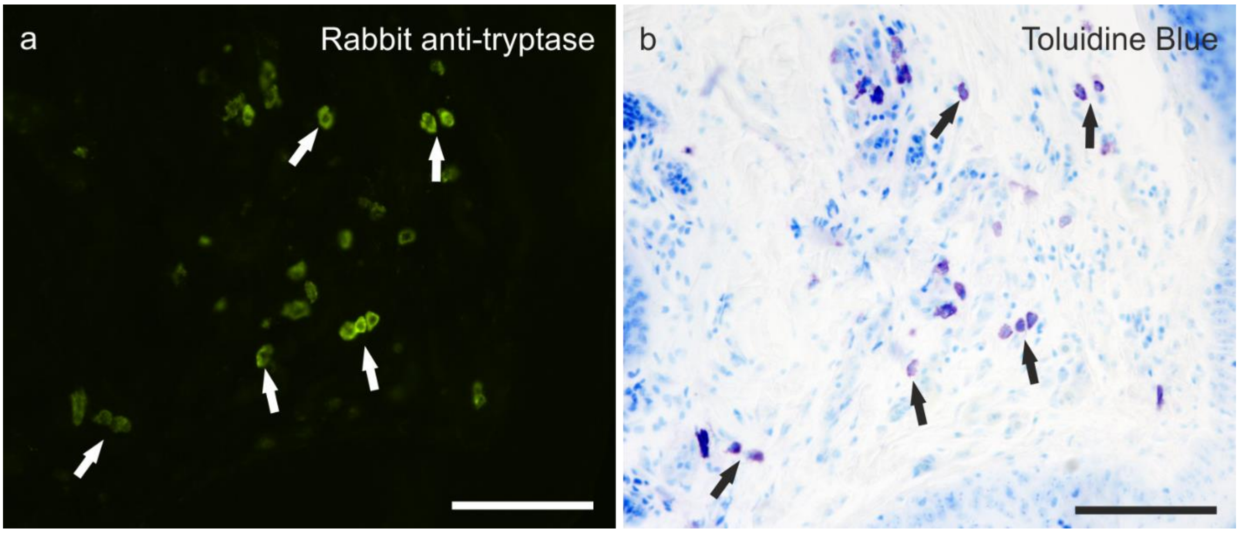

Supplementary Fig. 2.

Photomicrographs of a cryosection of canine skin on which the immunohistochemical staining (a) was associated with toluidine blue counterstain (b). The arrows indicate some mast cells identified with the polyclonal antibody (PAB070Ca01) against tryptase (a) and toluidine blue (b).

Bar: 50 µm.
