# Supplementary material for: Cannabinoid receptors in the inflammatory cells of canine atopic dermatitis
Source: Front Vet Sci. 2022 Sep 15;9:987132. doi: 10.3389/fvets.2022.987132 (PMC9521433; doi:10.3389/fvets.2022.987132)
Supplement: Supplementary file 3 [file Data_Sheet_3.PDF]

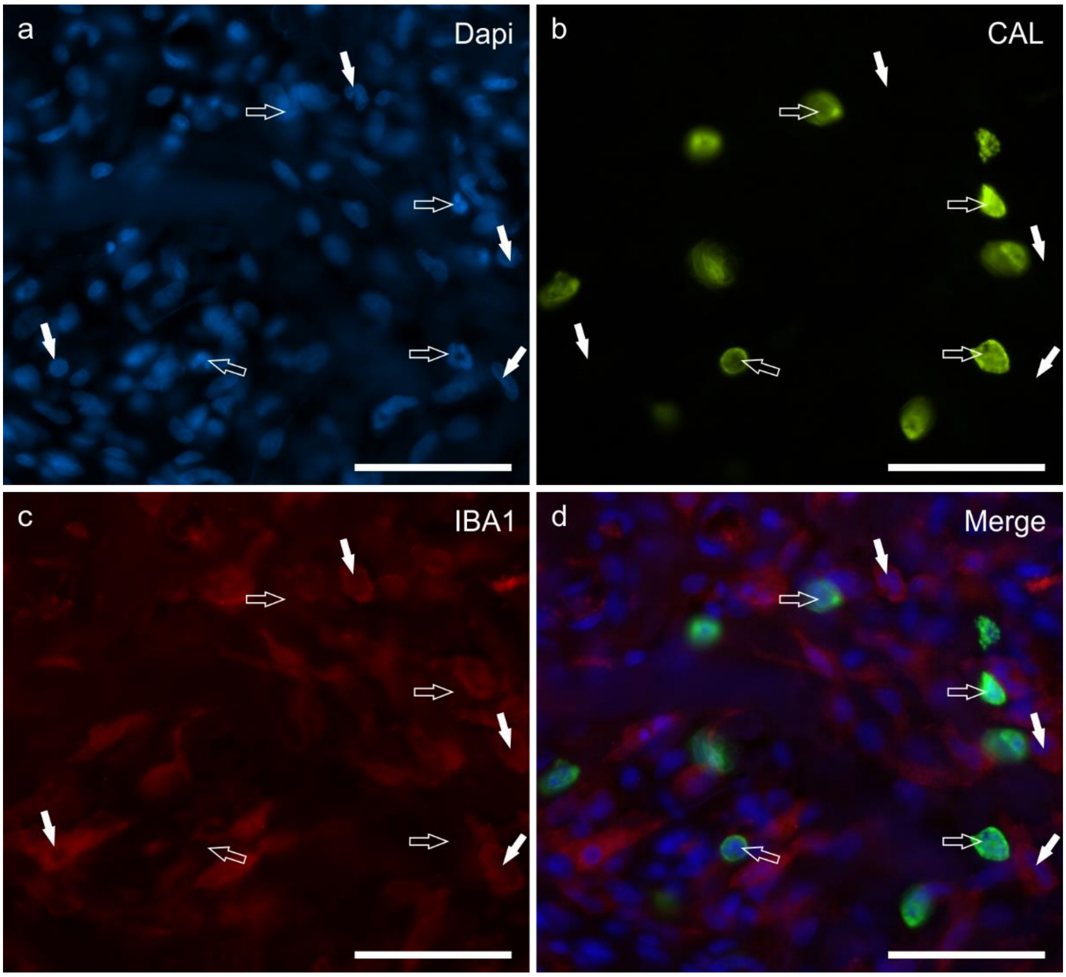

Supplementary Fig. 3.

Photomicrographs of a cryosection of the skin of a dog with atopic dermatitis on which the anti-calprotectin (CAL) (b) antibody (clone MAC387) was co-localized with the antibody anti-IBA1 (a macrophages marker) (c). The white arrows indicate the dapi-labelled nuclei of some IBA1 immunoreactive macrophages which were CAL negative. The open arrows indicate the Dapi-labelled nuclei of some CAL immunoreactive inflammatory cells which were IBA1 negative. The CAL immunoreactive cells showed multilobed nuclei, evidence which indicates that, at least in canine skin, the anti-CAL antibody mainly recognizes neutrophils.

Bar: 50 µm.
