# Supplementary material for: Cannabinoid receptors in the inflammatory cells of canine atopic dermatitis
Source: Front Vet Sci. 2022 Sep 15;9:987132. doi: 10.3389/fvets.2022.987132 (PMC9521433; doi:10.3389/fvets.2022.987132)
Supplement: Supplementary file 4 [file Data_Sheet_4.PDF]

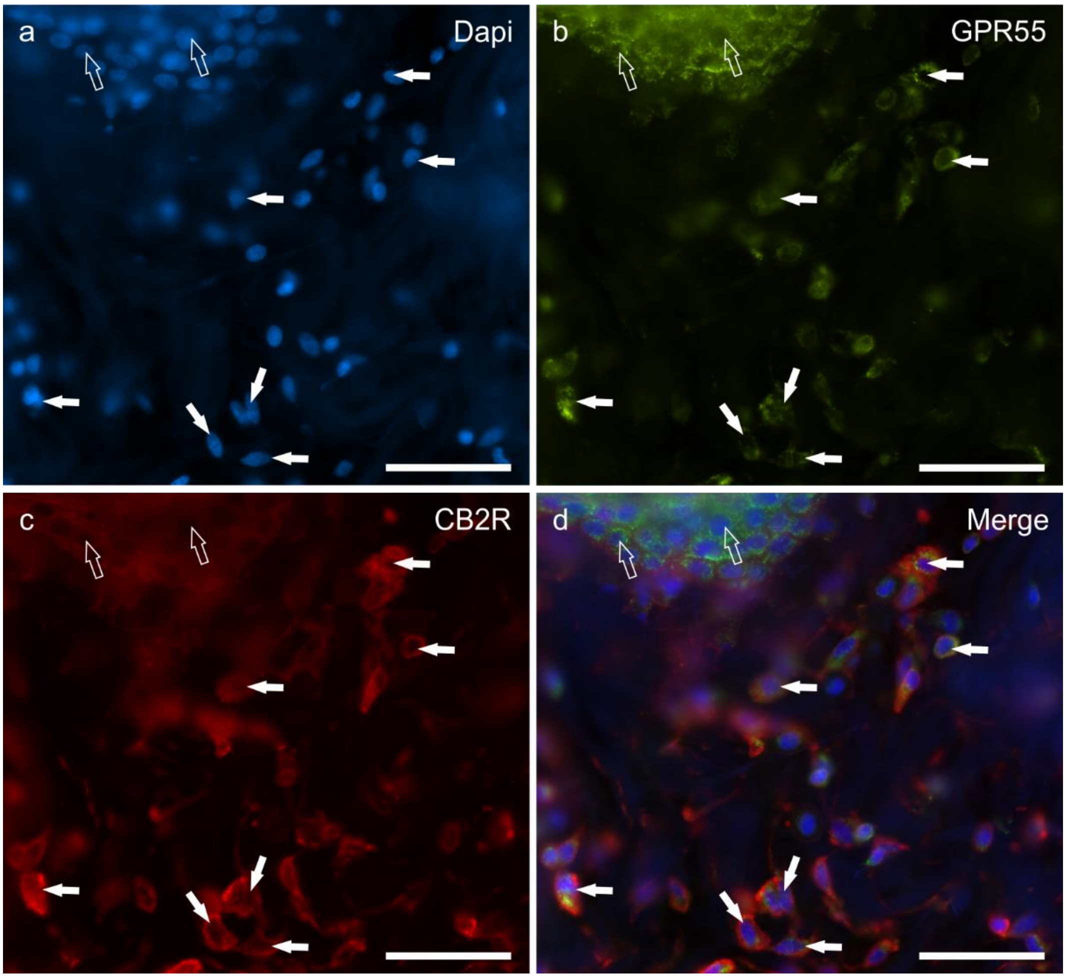

Supplementary Fig. 4.

Photomicrographs of a cryosection of the skin of a dog with atopic dermatitis on which the antibody anti-G protein-coupled receptor 55 (GPR55) (b) was co-localized with the antibody anti-cannabinoid receptor 2 (CB2R) (c). The white arrows indicate the Dapi-labelled nuclei of some inflammatory cells of the dermis co-expressing GPR55 and CB2R immunoreactivity. The open arrows indicate the dapi-labelled nuclei of epidermal cells (keratinocytes) expressing both the markers (GPR55 and CB2R).

Bar: 50 μm.
